# Genomic mapping of the MHC transactivator CIITA using an integrated ChIP-seq and genetical genomics approach

**DOI:** 10.1186/s13059-014-0494-z

**Published:** 2014-10-31

**Authors:** Daniel Wong, Wanseon Lee, Peter Humburg, Seiko Makino, Evelyn Lau, Vivek Naranbhai, Benjamin P Fairfax, Kenneth Chan, Katharine Plant, Julian C Knight

**Affiliations:** Wellcome Trust Centre for Human Genetics, University of Oxford, Roosevelt Drive, Oxford, OX3 7BN UK; William Harvey Research Institute, Barts and the London School of Medicine and Dentistry, Queen Mary University London, London, UK

## Abstract

**Background:**

The master transactivator CIITA is essential to the regulation of Major Histocompatibility Complex (MHC) class II genes and an effective immune response. CIITA is known to modulate a small number of non-MHC genes involved in antigen presentation such as *CD74* and *B2M* but its broader genome-wide function and relationship with underlying genetic diversity has not been resolved.

**Results:**

We report the first genome-wide ChIP-seq map for CIITA and complement this by mapping inter-individual variation in CIITA expression as a quantitative trait. We analyse CIITA recruitment for pathophysiologically relevant primary human B cells and monocytes, resting and treated with interferon-gamma, in the context of the epigenomic regulatory landscape and DNA-binding proteins associated with the CIITA enhanceosome including RFX, CREB1/ATF1 and NFY. We confirm recruitment to proximal promoter sequences in MHC class II genes and more distally involving the canonical CIITA enhanceosome. Overall, we map 843 CIITA binding intervals involving 442 genes and find 95% of intervals are located outside the MHC and 60% not associated with RFX5 binding. Binding intervals are enriched for genes involved in immune function and infectious disease with novel loci including major histone gene clusters. We resolve differentially expressed genes associated in *trans* with a *CIITA* intronic sequence variant, integrate with CIITA recruitment and show how this is mediated by allele-specific recruitment of NF-kB.

**Conclusions:**

Our results indicate a broader role for CIITA beyond the MHC involving immune-related genes. We provide new insights into allele-specific regulation of CIITA informative for understanding gene function and disease.

**Electronic supplementary material:**

The online version of this article (doi:10.1186/s13059-014-0494-z) contains supplementary material, which is available to authorized users.

## Background

The transcriptional regulation of the *CIITA* gene (also referred to as *C2TA* or *MHC2TA*) on chromosome 16p13, and the impact of genetic variation involving this gene for disease, has been extensively characterised following the identification of rare loss-of-function mutations in *CIITA* resulting in the bare lymphocyte syndrome and severe immunodeficiency due to lack of expression of Major Histocompatibility Complex (MHC) class II genes [1]. *CIITA* was found to encode a critical non-DNA binding factor, the master MHC class II transactivator, which is recruited to the class II enhancer complex and plays a critical role in expression of MHC class II genes and, as a result, in the adaptive immune response through antigen presentation to CD4+ T cells [2].

*CIITA* is expressed in a variety of antigen presenting cells both constitutively and following induction, notably after interferon-gamma (IFNγ), with transcriptional regulation of *CIITA* found to be complex and highly context specific. This includes the occurrence of four different promoters, each with a unique first exon, conferring considerable cellular specificity with, for example, the *CIITA* class III promoter important for constitutive expression in B cells while the class IV promoter is critical to inducible expression [2-5]. A number of enhancer elements have also been identified, including at least five elements over a 110 kb region spanning the gene [6].

CIITA regulates MHC class II gene expression through complex mechanisms including chromatin remodelling, transcriptional initiation and elongation [2]. However it does not directly bind DNA. Rather, it is recruited to the proximal promoter regions of the classical MHC class II genes (*HLA-DP*, *HLA-DR* and *HLA-DQ*), and to *HLA-DM*, *HLA-DO* and the *CD74* gene (encoding the molecular chaperone invariant chain which associates with the MHC class II complex) through protein-protein interactions with other components of the MHC class II enhanceosome. These include the regulatory factor X complex (RFX5, RFXANK and RFXAP), the cAMP responsive element binding protein (CREB1) and activating transcription factor 1 (ATF1), and nuclear factor Y (NFYA/B/C subunits) which bind DNA through the SXY module, a highly constrained series of sequences (S-X-*X*2-Y boxes) spanning 59 to 68 bp and typically located within 300 bp of the site of transcriptional initiation [2,7,8]. In addition, more distal SXY modules with enhancer activity have been identified within the MHC class II region [9]. SXY modules are also found in the MHC class I region where CIITA is recruited but has an ancillary role, the major trans-activator here being NLRC5 [10]. In addition to *CD74* and *B2M*, a small number of non-MHC target genes for CIITA have been identified following chromatin immunoprecipitation (ChIP) for CIITA analysed using promoter arrays [8] and differential gene expression in cell lines deficient in CIITA [11] including *RFX5*, *TPP1*, *RAB4B*, *PSMD3* and *MYBPC2* which were found to depend on RFX recruitment to a promoter X-box sequence.

Genome-wide functional genomic approaches provide new opportunities to systematically define such regulatory elements and the impact of genetic variation. Here we describe the first ChIP-seq derived genome-wide map for CIITA occupancy, set in the context of complementary data for other DNA-binding protein members of the CIITA enhanceosome and regulatory features of the epigenomic landscape. We demonstrate how this can be integrated with data mapping the genetic determinants of inter-individual variation in CIITA expression, resolving associated target genes for CIITA both within the MHC and in the larger genomic space outside the MHC. We show how a specific intronic regulatory variant of *CIITA* is associated in *trans* with a network of target genes and modulates allele-specific recruitment of NF-KB.

## Results

### A genome-wide map of CIITA recruitment in human B cells and monocytes

In order to generate a high-resolution genome-wide map of CIITA recruitment, we performed ChIP for CIITA followed by high throughput sequencing (ChIP-seq) in primary human B cells and monocytes. Published work to date suggests that CIITA binding is associated with RFX binding to an X box sequence and our experimental design included generating ChIP-seq data for CIITA and RFX5 using chromatin prepared from the same cells. We analysed CD19+ B cells, which show high constitutive levels of MHC class II expression, together with CD14+ monocytes either resting or treated with IFNγ as an inducible paradigm for MHC class II expression, as this cytokine is known to significantly upregulate gene expression [12].

**Figure 1:**
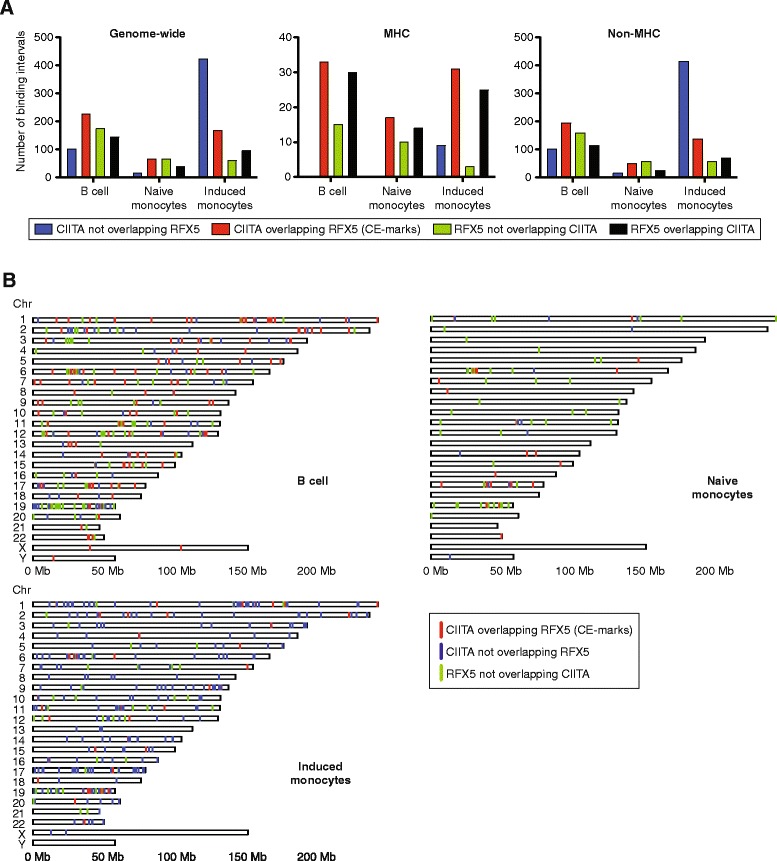
**CIITA and RFX5 binding in B cells, naïve and IFNγ treated monocytes. (A)** Numbers of CIITA and RFX5 BIs identified as occurring alone or overlapping. Counts shown genome-wide, within the MHC or excluding the MHC (MHC region defined as chr6: 29,580,000-33,100,000). **(B)** Chromosomal location of CE-marks and of non-overlapping CIITA and RFX5 BIs.

ChIP-seq peaks for CIITA and RFX5 were scored using the model-based analysis of ChIP (MACS) after mapping to the reference human genome haplotype sequence for the MHC region (chr6: 29,580,000-33,100,000). We defined a set of 1,010 binding intervals (BIs) genome-wide for CIITA and RFX5 across 12 samples derived from highly purified CD19+ B cells and CD14+ monocyte populations from two different healthy individuals (Figure 1A and Additional file 1: Table S1). Within a given cell type or condition, BIs were defined as CIITA and/or RFX5 enriched ChIP-seq peaks present in both individuals. When we performed an analysis of CIITA and RFX5 affinities (differences in read densities) across the BIs, this partitioned the binding profiles for B cells and monocytes into two distinct groupings consistent with cell type (Additional file 2: Figure S1A). For CIITA, we identified 329 BIs in B cells, 81 in resting monocytes and 591 in monocytes treated with IFNγ. Specific known and novel CIITA BIs were validated by ChIP for a third individual using a different CIITA antibody (Additional file 2: Figure S2). We integrated all CIITA and RFX5 BIs across the 12 ChIP-seq datasets (Additional file 1: Table S1) with DNase I hypersensitivity site (DHS) maps for primary B cells and monocytes [13]. This revealed that genome-wide, 95% of CIITA BIs in B cells and 79% in naïve monocytes are co-incident with a DHS (Additional file 1: Table S1). We investigated the overall relationship with components of the canonical CIITA enhanceosome RFX5, CREB1, ATF1, NFYA and -YB for CIITA BIs. The majority of BIs, 81% and 98% for B cells and naïve monocytes, respectively, have data coincidence scores (DCS) greater than 4 (Additional file 1: Table S1 & Additional file 2: Figure S1B) suggesting that genome-wide, CIITA occupancy involves sites of accessible chromatin and that other constituents of the canonical enhanceosome may be relevant to recruitment.

We proceeded to characterise further the relationship of CIITA and RFX5, defining instances where CIITA BIs are co-incident with RFX5 binding as CIITA Enhanceosome marks (CE-marks) (Figure 1A and B). In primary human CD19+ B cells, 329 CIITA and 318 RFX5 BIs were mapped genome-wide (Additional file 1: Table S1) and 69% of CIITA BIs overlapped with RFX5 to form CE-marks (Figure 1A). Within the classical MHC Class II region (chr6: 32,400,000-33,100,000), we found all CIITA BIs were CE-marks. For primary CD14+ monocytes in a naïve state, there were 81 CIITA and 105 RFX5 BIs of which 81% overlap to form CE marks (Figure 1A) while within the MHC Class II region, there were 17 CIITA BIs that, as observed with B cells, were all CE marks. When monocytes were treated with IFNγ, we observed 591 CIITA BIs (a 7.3-fold increase compared to the naïve state) with 28% of CIITA BIs being co-incident with RFX5 and forming CE marks. Within the MHC Class II region the proportion of CE marks increased to 78% and of these, 7/31 were stimulus specific. In contrast, all CIITA BIs within the MHC Class II region of naïve monocytes were also present in treated cells. Similarly, outside of the MHC we observed a lower proportion of CIITA binding in naïve monocytes as compared with induced (Figure 1A). We observed that some CE marks were identified across all cell types and conditions both within and outside of the MHC region. Overall, we find that for B cells and resting monocytes all CIITA BIs within the MHC class II region were CE marks while only 78% were CE marks following IFNγ treatment of monocytes (Figure 1A). We found that outside the MHC region, in B cells and naïve monocytes 66% and 77%, respectively, of CIITA BIs were CE marks while after IFNγ treatment only 25% of CIITA BIs in monocytes were identified as CE marks.

**Figure 2:**
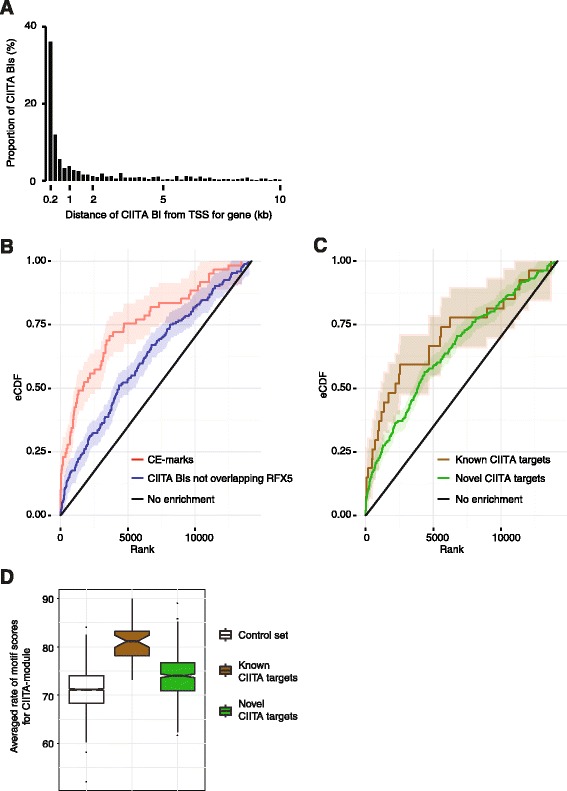
**CIITA BIs in relation to gene location, CIITA module and gene function. (A)** Distribution of CIITA BIs showing relative distances from the TSS of genes. **(B)** Enrichment of genes that were differentially expressed in treated monocytes within two groupings of CIITA target genes, one associated with CE marks (CIITA BIs overlapping RFX5) and the other CIITA BIs without RFX5. The empirical cumulative distribution functions (eCDFs) for each grouping is shown with a 95% confidence interval. A group of CIITA targets containing a larger than expected number of differentially expressed genes will produce an eCDF that lies above the line corresponding to absence of enrichment. **(C)** Enrichment for all CIITA BIs (overlapping and non-overlapping with RFX5) associated with known and novel CIITA target genes in treated monocytes. **(D)** Box plot showing averaged rate of MATCH-PWM binding site scores for a CIITA module of RFX5, CREB and NF-Y motifs (co-factors of the classical CIITA enhanceosome) in promoters of known CIITA target genes and those identified in this study (denoted as novel targets where CIITA BIs localised within 10 kb 5′ of the TSS). For known and novel target genes, 150 bp intervals centred on CIITA peak summits were analysed. A control set comprised of 1,000 randomly selected segments is included for comparison. Distributions of the three sets were compared using Mann-Whitney-Wilcoxon test, showing that these are significantly different, between the control set and known CIITA targets (*P* = 2.2 × 10^-16^) or novel targets (*P* = 1.5 × 10^-11^), and between known and novel targets (*P* = 2.7 × 10^-7^).

We next considered the relationship of CIITA BIs with genes and gene networks. We defined a potential functional relationship with a gene when the distance of a CIITA BI from the transcriptional start site (TSS) of a gene was <10 kb (5′ or 3′). Following this definition, we found that 635 of 843 CIITA BIs (75%) were associated with a gene (Figure 2A), with a total of 442 genes identified (Additional file 1: Table S1). CIITA BIs in B cells, naïve monocytes and monocytes treated with IFNγ showed association with 242, 40 and 271 genes, respectively (Additional file 1: Table S1). Across all cell types and conditions, a single BI was found within 2 kb of the TSS for 75% to 94% of these genes. For a further 3% to 16% of these genes a single, more distal BI within 10 kb of the TSS was present. Finally, for the remaining 5% to 9% of these genes there was association with both proximal and more distal BIs.

We identified networks enriched for genes associated with a CIITA BI for genome-wide data using DAVID (the database for annotation, visualization and integrated discovery) bioinformatics resources [14,15] and Ingenuity Pathway Analysis tools. The enriched gene networks revealed biological themes related to immunity and inflammation, with antigen presentation the most significantly enriched pathway on global analysis of all CIITA BI associated genes (*P* = 4.7 × 10^-19^) and when CIITA BIs identified in each of the specific cell types were analysed (Additional file 1: Table S1). Signalling involving OX40, a member of the TNFR superfamily important in T cell activation, was also significantly enriched on analysis of all CIITA BI genes (*P* = 1.8 × 10^-18^) and for individual cell types. In terms of disease, we found that autoimmune disease phenotypes were significantly enriched across different cell types in the CIITA BI associated genes. In B cells, RFX5 was the most significant predicted upstream transcriptional regulator (*P* = 1.4 × 10^-9^) together with NLRC5 (*P* = 3.6 × 10^-8^), SRF (*P* = 3.8 × 10^-6^) and CIITA (*P* = 4.4 × 10^-5^) while in naïve monocytes RFX5 (*P* = 4.6 × 10^-9^) and CIITA (2 × 10^-7^) were most significant (Additional file 1: Table S1). In induced monocytes, a more complex picture was seen with STAT1 (*P* = 9.3 × 10^-12^), NFkB complex (*P* = 1.5 × 10^-10^) and IRF1 (P = 1.1 × 10^-9^) identified as upstream regulators together with RFX5 (*P* = 3.3 × 10^-9^).

We proceeded to examine for possible enrichment of genes associated with a CIITA BI among genes that are differentially expressed in monocytes after IFNγ treatment [16]. We found significant enrichments of such genes, with highest enrichment when a CE mark was present but there was also an enrichment of genes associated with a CIITA BI where RFX5 was absent (Figure 2B). There was a similar enrichment observed when genes associated with CIITA BIs (combined set of CE marks and CIITA BIs without RFX5) were segregated into known CIITA targets (Additional file 1: Table S1) and novel CIITA targets identified in this study (Figure 2C). At the same time, we also observed that there were other instances whereby differential gene expression between naïve and treated monocytes we previously defined in a large cohort [16] were not associated with a change in CIITA occupancy (Additional file 2: Figure S3).

### Resolving proximal and distal sites of CIITA recruitment in the MHC class II region

**Figure 3:**
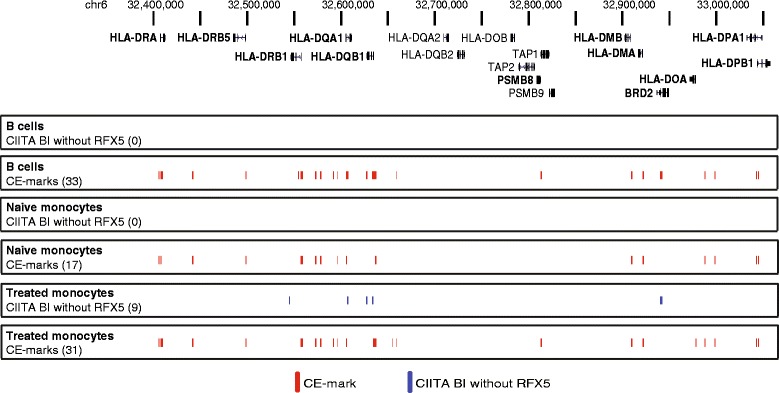
**Location of CIITA BIs and CE marks within the MHC Class II region (chr6: 32,400,000-33,100,000).** Numbers of CIITA BIs inclusive of CE marks are shown in parentheses.

CIITA is known to be recruited to the enhanceosome complex at proximal promoter SXY sequences of classical MHC class II genes with a small number of more distal enhancer elements recruiting CIITA identified based on sequence homology with the SXY module [9]. Within the MHC class II region we identified a total of 30 CIITA BIs across freshly isolated B cells and monocytes. The majority (73%) of these CIITA BIs were located within 10 kb of the TSS including classical genes such as *HLA-DP*, *HLA-DR* and *HLA-DQ* (Figure 3). Furthermore, for all of these genes there was a CIITA BI located proximally within 2 kb of the promoter (Additional file 1: Table S1). We observed that IFNγ treatment in monocytes resulted in nine additional CIITA BIs not present in naïve cells (8 proximal; 1 distal) that were associated with *BRD2*, *PSMB8*, *HLA-DRA*, -*DQA*, -*DQB* and *-DOA* (Figure 3).

**Figure 4:**
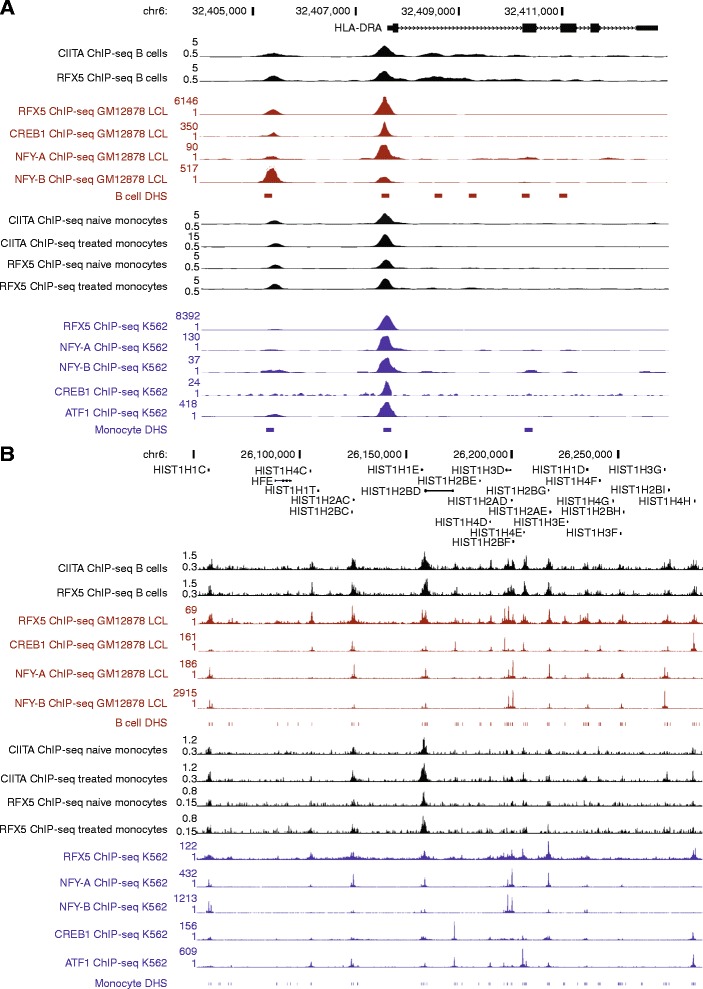
**CIITA binding intervals for a classical gene within the MHC class II region and in a histone cluster 1. (A)** Identification of two CIITA BIs, one proximal and other distal to the TSS of *HLA-DRA*. Both are CE marks and binding is associated with other constituents of the CIITA Enhanceosome (NFYA/ -YB; ATF1 and CREB1) and are positioned within DNase I hypersensitive sites. **(B)** Multiple CIITA BIs within a major histone cluster at chr 6p21.3.

We resolved proximal and distal CIITA recruitment within the MHC class II region as illustrated by the following examples. For *HLA-DRA*, we found evidence of a proximal and a more distal CIITA BI (Figure 4A) with the latter corresponding to a known site for CIITA occupancy [9]. In this instance, both CIITA BIs were associated with RFX5 binding (CE marks), located within transcriptionally accessible regions (DNaseI DHS) and associated with other features of the MHC enhanceosome including binding by CREB1, ATF1, NFYA and -YB. We mapped CIITA recruitment to the proximal promoter regions of *HLA-DRB1* and *HLA-DQA1* and identified a further four CIITA BIs between these genes (Additional file 2: Figure S4). These included a CIITA BI localising to an isoform specific promoter for *HLA-DQA1* (chr6: 32590730-32591333); a CIITA BI localising to the XL9 intergenic regulatory element previously reported to bind CIITA while also recruiting CTCF to enable chromatin looping and modulation of *HLA-DRB1* and *HLA-DQA1* gene expression (chr6: 32595564-32596093) [17]; a previously reported CIITA BI (chr6: 32577249-32578269) [9]; and a novel CIITA BI (chr6: 32571777-32572798) that has not been previously described. All were CE marks in at least a single cell type and condition.

We also identified a distal CIITA BI in an intergenic region 50.9 kb 5′ of *HLA-DQA2* (chr6: 32657986-32658366). This site has previously been reported as a target of CIITA and RFX5 [9] and we found evidence of a CE mark in B cells with inducibility in monocytes treated with IFNγ. Finally, we resolved in the region spanning *HLA-DOA* and *HLA-DPA1* that there were two proximal promoter CIITA BIs and a further two more distal CIITA BIs (chr6: 32986547-32987178 and chr6: 32996961-32997920), both of which were also CE marks. The former is novel while the latter has been reported to be a target of CIITA and RFX5 [9].

Within the MHC class I region (chr6: 29640000-31325000), where CIITA plays an ancillary role [10], we identified 11 CIITA BIs of which 10 are CE marks (Additional file 1: Table S1). These included the proximal promoter regions of classical class I genes including *HLA-A*, *HLA-B*, *HLA-C*, *HLA-E* and *HLA-F*. In addition we found evidence of CE marks at *TRIM26* in B cells and monocytes, consistent with earlier reports [8], and novel recruitment at the proximal promoter of *PPP1R10* in B cells (Additional file 2: Figure S5). *PPP1R10* encodes the protein phosphatase I regulator PNUTS, recently shown to play a key role in DNA damage repair [18].

### Non-MHC CIITA recruitment

We observed that of 843 CIITA BIs identified across B cells and monocytes, the majority were located outside of the MHC (90% of CIITA BIs in B cells, 79% in naïve monocytes, 93% in treated monocytes). For these CIITA BIs, 40% were associated with RFX5 binding and classified as CE marks. As observed within the MHC, the majority of CIITA BIs associated with a gene (<10 kb from TSS) were similarly localised in terms of proximity to the TSS (70% are within 2 kb of the TSS compared to 82% within the MHC class II region; Figure 2A and Additional file 2: Figure S6) and within transcriptionally accessible regions of the genome (Additional file 2: Figure S1B and Additional file 1: Table S1).

Our global analysis of genes associated with CIITA BIs highlighted gene networks related to immunity and inflammation (Additional file 1: Table S1). Considering all CIITA BIs we identified outside the MHC, we found greater diversity in the associated pathways with EIF2 signalling (*P* = 4.5 × 10^-10^) most significant while IL17A and JAK/STAT signalling were also enriched. Cell proliferation (*P* = 7.3 × 10^-12^) and RNA expression (*P* = 4 × 10^-11^) were significantly enriched in terms of biological process. Striking enrichment was also seen for viral infection (*P* = 4.5 × 10^-11^) involving 82 genes together with a number of disease phenotypes involving arthritis (Additional file 1: Table S1).

CIITA binding has been previously reported for regions outside the MHC, notably *CD74* and *B2M* which both encode key components found in MHC class II and class I molecules, respectively [8,19]. Our ChIP-seq maps resolved a CE mark located proximally to the TSS of *CD74* on chromosome 5 (Additional file 1: Table S1 and Additional file 2: Figure S7). CIITA occupancy at this location has been previously described by Krawczyk and co-workers [8]. There was also a CE-mark located at the proximal *B2M* promoter (Additional file 1: Table S1 and Additional file 2: Figure S8). Our datasets also confirmed previously reported CIITA targets [8] with CIITA BIs including CE marks identified at *RFX5, TPP1*, *RAB4B*, *ZNF672, HAUS5* and *MYBPC2/SPIB*. These reported targets had lower *P* values for the confidence of calling peak summits (Additional file 2: Figure S9).

In both B cells and treated monocytes, we observed the presence of multiple histone genes within the enriched gene networks (Additional file 1: Table S1). These included the histone H3.3B gene *H3F3B* for which a CE mark can be found at the proximal promoter (chr17: 73775313-73776112). Within the large histone gene cluster at 6p21.3-6p22, we identified a further 11 CIITA BIs encompassing four CE marks corresponding to proximal promoter regions of HIST1 genes including *HIST1H1C, HIST1H2BC, HIST1H2BD, HIST1H4D, HIST1H2AD, HIST1H4E, HIST1H2BG, HIST1H2BI* and *HIST1H4H* (Figure 4B). We found another grouping of CIITA BIs including CE marks at a second major histone gene cluster with *HIST2H2AB* and *HIST2H2BE* at chr1q21.2. In B cells, we identified a CE mark at the proximal promoter of *HIST4H4* (Additional file 1: Table S1).

Given that 60% of CIITA BIs identified outside of the MHC were not overlapping RFX5 BI, we sought to further characterise CIITA recruitment and possible associated co-factors. We first investigated the occurrence of the tri-motif CIITA module comprised of RFX5, CREB and NF-Y [20] within 150 bp of sequence around peak summits for CIITA BIs localised to within 10 kb upstream of a TSS. The module was most prevalent for CIITA BIs localised at known CIITA targets that include genes within the MHC Class II region (Figure 2D and Additional file 1: Table S1). The module was also enriched within the novel CIITA BIs identified in this study but significantly less than observed for CIITA BI involving known loci.

We then investigated occurrence of known DNA binding motifs for other transcription factors where CIITA recruitment was occurring in the absence of RFX5 binding. We analysed the 150 bp sequences spanning CIITA peak summits for enriched consensus sequences using the MEME-ChIP suite [21,22]. This showed significant enrichment of several consensus motifs notably STAT3 and SPI1 in IFN-treated monocytes (Additional file 1: Table S1).

To further investigate this, we analysed upstream regulators of genes associated with CIITA BIs not overlapping RFX5 BI using IPA (Additional file 1: Table S1). Analysis of all genes associated with CIITA BIs revealed that while CE marks were most significantly associated with RFX5 (*P* = 4.5 × 10^-10^) and NLRC5 (*P* = 1.4 × 10^-8^), CIITA BI not overlapping RFX5 were significantly enriched for transcription factors STAT1 (*P* = 2.2 × 10^-7^), JUN (*P* = 8.7 × 10^-7^), MYC (*P* = 8.9 × 10^-7^), STAT3 (*P* = 5.4 × 10^-6^) and NFkB complex (7.5 × 10^-6^). A similar pattern was seen when genes associated with CIITA BI in the MHC were compared with non-MHC genes. Considering specific cell types, this effect was most clearly seen on analysis of induced monocytes (Additional file 1: Table S1) with STAT1 (*P* = 2.3 × 10^-8^), NFkB complex (*P* = 2.8 × 10^-7^) and STAT3 (*P* = 3.8 × 10^-7^) the most significantly enriched transcriptional regulators in CIITA BI not overlapping RFX5.

The motif analysis and upstream regulator data raise the hypothesis that members of the STAT family may be involved in CIITA recruitment in the absence of RFX5 but further work is required to test this. In terms of diseases and functions, the overall gene set we identified as associated with CIITA BIs not overlapping RFX5 was most significantly enriched for infectious disease (viral infection) (*P* = 1.6 × 10^-8^) followed by a variety of autoimmune diseases with effects most clearly seen in treated monocytes. In B cells and naïve monocytes enrichment for cancer phenotypes was noted, with death receptor signalling identified on pathway analysis in B cells (Additional file 1: Table S1).

### Insights from the genetical genomics of *CIITA*

Mapping gene expression as a quantitative trait has the potential to define master regulatory networks through resolution of *trans* associations in which differential gene expression for a gene set are seen to be associated with a specific genetic marker [23]. We have reported genome-wide expression quantitative trait (eQTL) analysis in different primary human cell populations [24]. This global analysis included evidence of a local, *cis*-eQTL involving *CIITA* that was specific to B cells. Given the role of CIITA as a master regulator we sought to further resolve this association and to investigate whether there was evidence of associated *trans*-eQTL networks that could inform CIITA function and modulated genes.

**Figure 5:**
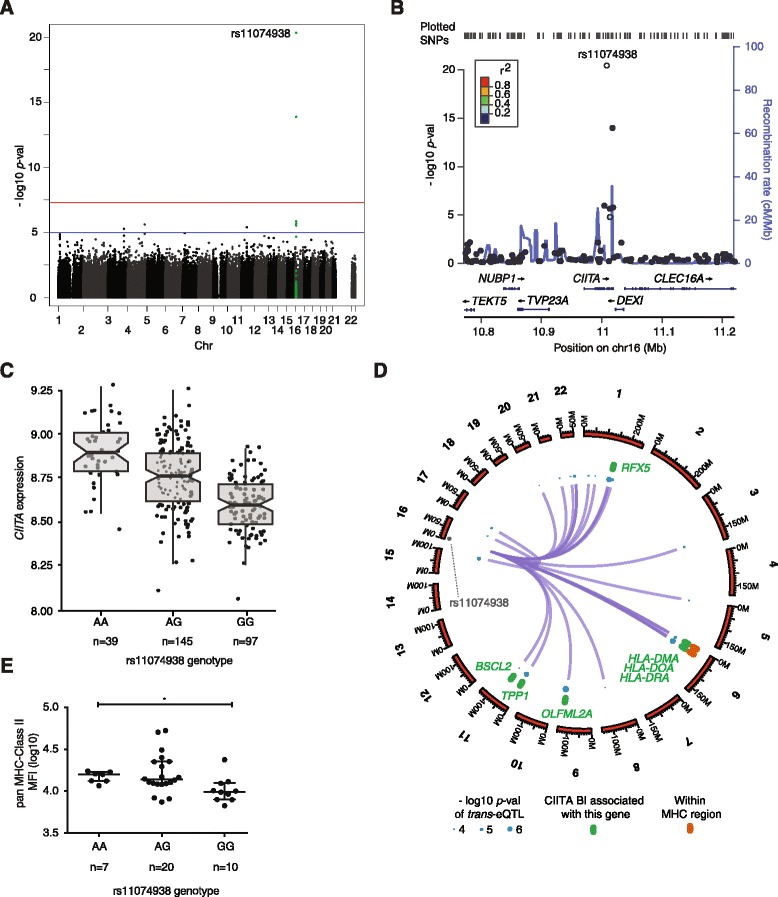
**Local and distant eQTL associations for eSNP rs11074938 involving** ***CIITA*** **. (A)** Manhattan plot showing association on chromosome 16p13. **(B)** Regional association plot for CIITA demonstrating peak association for rs11074938 located in intron 13 of *CIITA*. **(C)** Box-plot showing allelic association of rs11074938 with levels of CIITA expression across 281 individuals. **(D)** Circular plot illustrating genomic location of eQTLs for rs11074938 in B cells. From outside to inside: chromosome number; relative distance along chromosome; coloured dots are *cis* and *trans*-eQTLs (size relative -log10 *P* value as per legend); the outer grey dot is a *cis*-eQTL for CIITA while inner blue dots are *trans*-eQTLs; lines indicate genes regulated in *trans* rendering these as potential CIITA targets, only eQTL mapping to genes with *P* <3 × 10^-4^ in the dataset (*n* = 281) have been shown. **(E)** Association of rs11074938 with levels of pan MHC class II protein across 37 individuals quantified using multi-parametric flow cytometry. Difference in measured protein levels between homozygous individuals with the A allele as compared to the G allele was significant (*denotes Kruskall-Wallis *P* = 0.02; MFI = Median fluorescence intensity; data shown have been normalised to control isotype staining).

We used a linear model which incorporated major principal components of the expression data as covariates [25] to further define association within 1 Mb of *CIITA* for the 281 healthy volunteers using a genome-wide panel of 651,210 SNP markers [24]. This resolved that the strongest association involved a single nucleotide polymorphism (SNP) rs11074938 in the 13th intron of *CIITA* (*P* = 6.9 × 10^-20^, FDR *=* 3.2 × 10^-15^) (Figure 5A to C). Analysis of *trans* associations involving this SNP (Figure 5D) revealed multiple associations with MHC Class II genes, notably *HLA-DMA*, *HLA-DOA* and *HLA-DRA* consistent with the known role of CIITA in regulation of these genes. Non-MHC *trans* associations included genes that are known to show evidence of CIITA recruitment such as *RFX5* and *TPP1* while for others including *OLFML2A* and *PARVG* we find ChIP-seq mapping supports a role for CIITA.

We sought to determine whether the *trans* association with transcript expression seen for rs11074938 with MHC Class II genes was also observed at the protein level. We assayed MHC class II surface expression on B cells in whole blood from healthy volunteers by multi-parametric flow cytometry and observed that individuals possessing one or two copies of the rs11074938 A allele compared to the G allele expressed higher levels of protein (Figure 5E).

### A cis-eQTL for *CIITA* involving allele-specific recruitment of NF-KB

**Figure 6:**
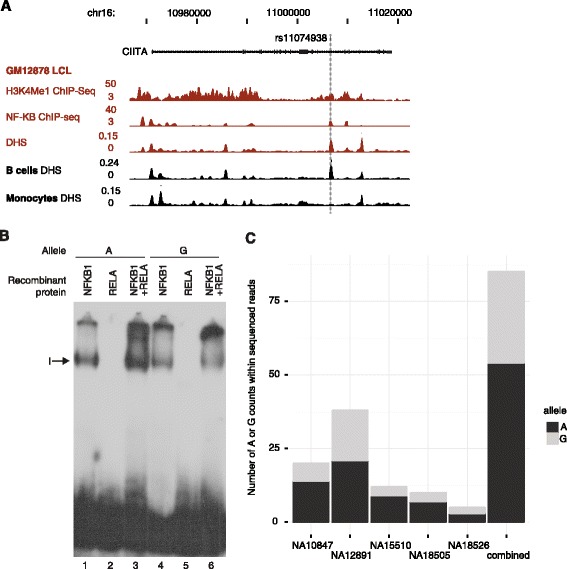
**Functional characterisation of** ***CIITA*** **regulatory variant. (A)** Genomic landscape of *CIITA* and the eSNP rs11074938. Data from ENCODE project for a lymphoblastoid cell line, GM12878 and primary CD20+ B cells and CD14+ monocytes. The SNP, rs11074938 is localised to a DHS specific to B cells that is further coincident with a NF-KB ChIP-seq peak. **(B)** EMSA demonstrating allele-specific binding for NF-KB involving rs11074938. Recombinant NF-KB proteins NFKB1 (lanes 1 and 4), RELA (lanes 2 and 5) or both (lanes 3 and 6) incubated with probes corresponding to the two different alleles for rs11074938, either A (lanes 1 to 3) or G (lanes 4 to 6). **(C)** Ratio of A and G alleles within sequenced reads spanning SNP rs11074938 from NF-KB ChIP-seq analyses of five heterozygous individuals.

In light of the *cis* and *trans* associations for rs11074938 in B cells, we sought to investigate the functional basis for this. We first studied the regulatory functional genomic landscape of the sequence variant. Analysis of publically available ENCODE data revealed that SNP rs11074938 was located within a DHS in human primary B cells and lymphoblastoid cell lines (LCLs) but not monocytes (Figure 6A). The position in a region of open chromatin was consistent with the hypothesis that the variant is located in a regulatory element. This was further supported by the presence of specific histone marks including H3K4Me1 and evidence that this is a phylogenically conserved region (vertebrate conserved element, LOD score 31).

Analysis of ChIP-seq data [26] revealed that the SNP was located within an NF-KB target site in LCLs (Figure 6A) and contained an NF-KB consensus binding motif. We investigated whether the A to G substitution could modulate predicted binding affinity using a publically available database identifying binding sites of NF-KB by a combination of protein-binding microarrays and surface plasmon resonance [27]. The ancestral A allele was predicted to show higher binding affinity for NFKB1/RELA (z-score human 3.25 with A allele vs. 2.83 with G allele) and NFKB2/RELB (z-score 3.16 A vs. <2 G) but the situation is complex with the G allele also predicted to show higher affinity for the NFKB2 homodimer (z-score 4.38 G vs. 3.86 A).

To investigate this further we characterised NF-KB binding by electrophoretic mobility shift assay (EMSA). EMSA using recombinant NFKB1 and RELA proteins demonstrated that with the NFKB1/RELA heterodimer there was greater binding to the A allele compared to the G allele (Figure 6B). Weak binding was observed for NFKB1 alone with no evidence of RELA binding as a homodimer.

We then proceeded to determine whether allelic differences in NF-KB binding were present *ex vivo* using ChIP-seq data for a panel of five LCLs heterozygous for rs11074938 [26]. This revealed that there was significantly more RELA binding to the A allele compared to the G, with on average 66.1% (SD 8.1) of reads mapping to the A allele (*P* =0.008) (Figure 6C).

In order to investigate whether the intronic sequence spanning the SNP rs11074938 had regulatory activity that could be modulated in an allele-specific manner, we cloned the region into a reporter gene construct. Given the low transient transfection efficiency of human B cell lines, we performed reporter gene assays in a lymphoblast cell line of T cell origin (Jurkat). This demonstrated evidence of increased enhancer activity for the A versus the G allele of rs11074938 with allele-specific differences in protein-DNA interactions also seen in this cell line (Additional file 2: Figure S10).

## Discussion

This work defines a high-resolution map of CIITA occupancy within the MHC and genome-wide, presenting evidence for complexity and specificity in the associated enhanceosome complex across different primary peripheral blood cell types and conditions. The results support a role for CIITA beyond the MHC that is often not associated with RFX5 binding but occurs in proximity to genes enriched for immune function and infectious disease, and highlights diverse potential roles including recruitment at histone gene clusters.

The biology of CIITA regulation for MHC class II genes is well established [2,7,8]. Our data support evidence for distal as well as classical proximal sites of CIITA recruitment [9], including novel BIs which, as elsewhere in the class II region, appear highly stereotyped in terms of enhanceosome composition and partner DNA binding proteins notably RFX5. Similarly, for the class I region the observed binding is consistent with previous findings at classical genes while supporting evidence for binding sites proximal to *TRIM26* and *PPP1R10* [10,28].

Genome-wide, the picture is more complex with 60% of observed CIITA BIs not associated with RFX5 binding based on ChIP-seq performed on the same input chromatin. As seen in the MHC, occupancy was associated with accessible transcriptionally active chromatin and was predominantly within 2 kb of a gene. The data confirmed some previously identified non-MHC genes such as *CD74* and *B2M* [8,11,19] but also highlighted many additional loci notably after monocytes were treated with IFNγ in which hundreds of additional BIs were revealed compared to the naïve state consistent with previous observations that CIITA-mediated regulation is intimately linked to treatment with interferons [2]. Examples of genes associated with CIITA BIs include key mediators such as *STAT1* and *STAT3*, observed specifically in monocytes treated with IFNγ and B cells, respectively, as well as regions of enrichment for CIITA BIs such as identified within the major histone gene clusters at chromosomes 6p21 and 1q21.

It has been proposed that in addition to its well-defined enhanceosome function in the MHC, CIITA may function to modulate transcription of a target gene through an alternative mode of action involving formation of a TFIID-like complex in conjunction with partners TAF6, TAF9 and TBP [19]. Within this complex, a TATA-box binding protein (TBP) is proposed to bind DNA and through the involvement of other chromatin modifying enzymes that bring about re-modelling of localised regions within the chromatin, CIITA within this TFIID-like complex may subsequently further dimerise with CIITA in a canonical RFX5-containing enhanceosome. The entire mechanism is however, currently not well understood.

We find that the well characterised, tri-motif CIITA module (RFX5, CREB and NF-Y) was less prevalent within novel CIITA BIs we identified but occurred more frequently than in a control set. Our analysis of peak summits within CIITA BIs not overlapping RFX5 highlighted enrichment of the STAT3 consensus DNA binding motif while STAT1 and STAT3 were significant predicted upstream transcriptional regulators for gene sets associated with such CIITA BIs. These data suggest a possible role for STAT family transcription factors in the recruitment of CIITA outside the MHC but further work is required to characterise this.

This work has highlighted the potential informativeness of integrating genomic profiling approaches such as ChIP-seq with *trans*-eQTL mapping to define modulated gene networks. We identified a strong local association with CIITA expression that we resolved to a specific *cis* acting expression-associated SNP (eSNP) involving allele-specific recruitment of NF-KB at an intronic regulatory element and differential gene expression. Further work is required to determine the detailed allele-specific transcriptional mechanisms involved in this *cis*-association. Strikingly, we find this eSNP was associated in *trans* with a gene network that included classical class II genes demonstrated at the transcript level and subsequently validated at the protein level. Moreover, the *trans* network also included non-MHC genes, both known targets such as *RFX5* and *TPP1,* and novel genes where we also find evidence of CIITA BIs by ChIP-seq. Our study had limitations in terms of sample size for determining a more extensive modulated gene network and further analysis at different time points following activation and CIITA expression are likely to be required for in-depth profiling and complete integration with maps of CIITA occupancy, together with functional evidence linking recruitment with differences in gene regulation. However the data illustrate that an integrated approach may be highly informative.

## Conclusions

Our study has demonstrated that CIITA recruitment commonly occurs outside the MHC, often not in the setting of a classical enhanceosome with RFX5, and involves genes enriched for immune function and infectious disease. We have functionally characterised a local regulatory variant of CIITA acting through allele-specific recruitment of NFkB which modulates MHC and non-MHC CIITA target genes in *trans*. The work provides a route-map for further studies to understand the role of CIITA in gene regulation genome-wide, defining complexity and cell type specificity as well as the potential utility of combining analysis of BIs through approaches such as ChIP-seq with experiments of a nature whereby gene networks may be identified through common variants modulating gene expression locally and at a distance through master regulators.

## Materials and methods

### Nuclear extracts, electrophoretic mobility shift assays (EMSA)

Recombinant NFKB1 (#31101) and RELA (#31102) proteins were obtained from Active Motif, UK. Nuclear extracts were prepared from Jurkat cells as previously described [29]. Oligonucleotide probes were radiolabelled with ^32^P-dCTP (Perkin Elmer, Beaconsfield, UK) and EMSA performed as previously described [30]. Probes were generated by annealing forward and reverse oligonucleotides spanning rs11074938 (A_for AGCTGAAAAGGGGAAATGTCTGAAA, A_rev AGCTTTTCAGACATTTCCCCTTTTC; G_for AGCTGAAAAGGGGGAATGTCTGAAA; G_rev AGCTTTTCAGACATTCCCCCTTTTC).

### Volunteer recruitment

The study was approved by the Oxfordshire Research Ethics Committee (COREC reference 06/Q1605/55) and volunteers of European ancestry were recruited in the Oxfordshire area following written informed consent through NIHR Oxford BioBank.

### Flow cytometry for analysis of HLA Class II expression

We assayed HLA class II expression by multi-parametric flow cytometry for whole blood from 37 healthy volunteers using a three-step procedure adapted from a protocol kindly shared by R. Apps from the National Cancer Institute [31]. Human IgG (Sigma-Aldrich, Dorset, UK) was added to each tube, followed by mouse anti-human antibodies directed against all class II alleles (clone Tu39 and SK10/SPV-L3 combined) or an isotype control (murine IgG, Sigma-Aldrich). After 20 min, cells were washed with Dulbecco’s phosphate buffered saline (DPBS, Sigma-Aldrich) and sheep IgG was added to each tube followed by sheep-anti-mouse PE (Sigma-Aldrich). After a further 20-min incubation at 4°C, cells were washed, secondary antibody sites were blocked with mouse IgG (Sigma-Aldrich) and mouse-anti-human CD19-FITC (clone J3-119, Beckman Coulter) was added. This suspension was incubated for 20 min at 4°C, cells were subsequently washed and red blood cells lysed with VersaLyse (Beckman Coulter, High Wycombe, UK) over 20 min, then washed and fixed with Reagent A (Life Technologies, Paisley, UK). Data were acquired immediately on a single FACSCanto (BD) machine using standardized settings verified for each data collection batch using BD seven-colour setup beads (BD). A minimum of 10,000 gated B cells were acquired for each sample (gating strategy illustrated in Additional file 2: Figure S11). The genotype of samples was anonymised during flow cytometry. Fluorescence-minus-one controls were used to set gate thresholds. Data were analysed in FLowJo (v10.0.6) and Graphpad Prism (v6). Data were normalised for isotype staining by simple subtraction of the isotype MFI in the PE channel from the HLA-II signal.

### Isolation of primary immune cells and ChIP-seq

Leukocyte cones derived from different anonymous individuals were obtained from the NHS Blood and Transplant Unit. These were processed using standard techniques as described by Neron and co-workers [32]. Briefly, a Ficoll gradient coupled with successive washes were used to isolate PBMCs from the cones [24]. Automated magnetic activating cell sorting, or autoMACS method (Miltenyi) was used to positively separate CD14+ and CD19+ cell populations starting from 500 million PBMCs following manufacturer’s instructions [33]. The technique routinely yields cell purity levels greater than 90% when cross-validated using a FACS-based assay. CD19+ cells were cross-linked for 10 min in 1% formaldehyde, while CD14+ cells were treated with 10 ng/mL IFNγ (R&D Systems) for 24 h alongside an untreated control before cross-linkage. Chromatin isolation, sonication using a BioRuptor (Diagenode) and immunoprecipitation using CIITA (Diagenode) and RFX5 (Santa Cruz) ChIP-grade antibodies were performed. Quality control of ChIP-ped DNA was achieved by quantification on a Qubit 2.0 fluorometer (Invitrogen) using the Quant-iT dsDNA HS Assay Kit (Invitrogen). In addition, real-time quantitative PCR was employed to determine enrichment at various genomic locations prior to sequencing (Additional file 2: Supplemental Methods). Approximately 5 to 10 ng of ChIP-ped DNA from each sample was processed into libraries, amplified and sequenced using 51-bp paired-end runs on the Illumina HiSeq (Additional file 2: Supplemental Methods).

### ChIP-ddPCR enrichment

Validation ChIP experiments with two CIITA antibodies (Diagenode C15410062 and SantaCruz sc-48797X) were performed using B cells and monocytes derived from an independent leukocyte cone. ddPCR was carried out on the QX-100 PCR system (Bio-Rad), using labelled probes and primer sets (sequences listed in Additional file 2: Supplementary Methods) obtained from MetaBion GmbH and following manufacturer’s procedures for ddPCR Supermix for Probes (Bio-Rad 186-3010). A duplexed configuration was employed with each well containing an assay for the sample (labelled in FAM) and a second assay for the Rab4A-control region (labelled in YakimaYellow). Enrichment of ChIP over input was determined after normalising for the ratio of corresponding measurements for the control region, Rab4A.

### Bioinformatic and statistical analysis including eQTL mapping, peak calling and derivation of BIs

Stampy version 1.0.21 [34] was employed to align 51 bp-reads to the Human genome UCSC hg19 (GRCh37) using the PGF haplotype as our reference for the MHC region. Processing and filtering of mapped reads were done using Bamtools version 2.3.0 and Samtools version 0.1.17 to remove duplicates, singletons and improperly mapped read pairs that included mappings to non-unique locations and discordant locations. Additionally, read pairs were removed when one or both reads had low mapping quality (<20). Across samples, 38% to 83% of read pairs remained after this filtering (Additional file 1: Table S1). Enriched peaks were determined with ‘Model-based analysis of ChIP’ (MACS version 2.0.10) by comparing ChIP samples to input samples with FDR <5%. Bedtools version 2.3.0 was used to identify regions shared across the 12 samples. Contiguous, shared regions were merged into a BI when there were enriched peaks for CIITA or RFX5 in both individuals within a single cell type/condition (Additional file 2: Figure S12). This generated 1,010 BIs representative of CIITA and RFX5 binding for further analysis. Plots generated based on BigWig representations of ChIP-seq data in the UCSC Browser show on the y-axis enrichment of peaks (ChIP versus input) with peak-enrichment values normalised using the SPMR (signal per million reads) function.

### Analysis of known CIITA module (RFX5, CREB and NF-Y) within CIITA BIs

Three distinct groupings of 150 bp sequence segments around peak summits were analysed: (1) two groups comprised of segments within CIITA BIs that are localised to within 10 kb upstream or 150 bp downstream of a TSS and that are either known CIITA targets (20 genes) or novel targets identified in this study (275 genes); (2) a control set comprised of 1,000 genes randomly selected from the genome with sequence extracted 10 kb 5′ and 150 bp 3′ of the TSS from which 150 bp segments randomly selected. The tool Biostrings 2.32.1 from the BioConductor Package was used to scan for presence of RFX5, CREB and NF-Y motifs (source for PWM-Position Weight Matrices used: JASPAR [35], Transfac [36] and Human Protein-DNA Interactome (hPDI) [37]) and score similarity of any motif identified within a segment against the PWM in question. This was carried out iteratively within a segment to obtain a CIITA module with motifs identified in the correct order and with the best score (highest similarity to PWM for all components). An average of scores for the components was computed and this is used as a measure of the prevalence of this known CIITA module within a CIITA BI.

### Motif search

Peak summits in BIs were used to identify enriched consensus sequences with known DNA-binding motifs using MEME-ChIP suite. The peak summits for each sample were extended to 75 bp-length sequences into both directions. Equivalent sized DHS in primary cells (ENCODE) were used for background estimation. A customised database containing 569 motifs was created using information from the JASPER and hPDI repositories. We estimated a new Markov model for background frequencies. Discovered motifs with similar E-values of less than 1.0 (default option) and *P* value of less than 0.01 to the known motifs were used for further study. To get motifs similar to known motifs, we searched ‘tomtom.txt’ output files for each sample and selected motifs occurring in both individuals in the same condition.

### Determining biological themes from genes that are regulated by CIITA

We annotated BIs within 10 kb from the TSS of the nearest gene using ChIPpeakAnno [38] and CisGenome [39]. Gene clusters formed using CIITA BIs from B cells, naïve and treated monocytes were analysed through the use of the DAVID Bioformatics Suite and Ingenuity Pathway Analysis (IPA, Ingenuity Systems [40]). IPA was used to define gene networks, significant upstream regulators and relationships with canonical signalling pathways.

### Enrichment of differentially expressed genes within CE-marks and CIITA BIs

To investigate whether genes associated with CIITA BIs in treated monocytes are enriched for genes that are differentially expressed when compared to naïve monocytes we used our published gene expression data [16]. Differential gene expression analysis was performed using the limma package. All genes with expression data were ranked by differential expression (*P* values). We then considered the distribution of ranks within two different sets of genes; genes associated with a CE mark and genes associated with CIITA BIs without RFX5. We also examined genes associated with CIITA BIs (includes CE marks and CIITA BIs without RFX5) that are either known CIITA targets (Additional file 1: Table S1) or novel targets identified in this study.

### Data access

ChIP-seq datasets have been deposited into the ArrayExpress database [41] under accession number E-MTAB-2424.

## Additional files

Additional file 1: Table S1 Worksheets S1.1-1.22. Additional file 2
**Supplemental Methods and Figures S1-12.**

## Abbreviations

ATF1: activating transcription factor 1
BI: binding interval
CE mark: CIITA Enhanceosome mark
ChIP: chromatin immunoprecipitation
ChIP-seq: analysis of ChIP by next generation sequencing
CREB1: cAMP responsive element binding protein
DAVID: database for annotation, visualisation and integrated discovery
EMSA: electrophoretic mobility shift assay
eQTL: expression quantitative trait
eSNP: expression-associated SNP
IFNγ: interferon-gamma
LCL: lymphoblastoid cell line
MACS: Model-based analysis of ChIP
MEME: Motif-based sequence analysis tools
MFI: median fluorescence intensity
MHC: Major Histocompatibility Complex
NFY: nuclear factor Y
SNP: single nucleotide polymorphism
SXY: S-X-*X*2-Y boxes
TBP: TATA-box binding protein
