# Supplementary material for: Genomic mapping of the MHC transactivator CIITA using an integrated ChIP-seq and genetical genomics approach
Source: Genome Biol. 2014 Oct 31;15(10):494. doi: 10.1186/s13059-014-0494-z (PMC4243378; doi:10.1186/s13059-014-0494-z)
Supplement: Additional file 2 — Supplemental Methods and Figures S1-12. [file 13059_2014_494_MOESM2_ESM.pdf]

# Supplementary materials

## Genomic mapping of the MHC transactivator CIITA using an integrated ChIP-seq and genetical genomics approach

Daniel Wong, Wanseon Lee, Peter Humburg, Seiko Makino, Evelyn Lau, Vivek Naranbhai, Benjamin P Fairfax, Kenneth Chan, Katharine Plant, Julian C Knight

## Contents

**Supplemental Methods**

**Supplemental Figures**

- **Supplemental Fig. S1.** CIITA and RFX5 binding affinities and coincidence with components of CIITA enhanceosome.
- **Supplemental Fig. S2.** Validation of CIITA ChIP.
- **Supplemental Fig. S3.** CIITA binding and gene expression in monocytes.
- **Supplemental Fig. S4.** Intergenic CIITA binding in the interval between *HLA-DRB1* and *HLA-DQA1* within the MHC Class II region.
- **Supplemental Fig. S5.** CIITA binding within the MHC Class I region at *PPP1R10*.
- **Supplemental Fig. S6.** Distribution of CIITA BIs localised within and outside of the MHC region in relation to relative distance from the TSS.
- **Supplemental Fig. S7.** CIITA binding outside of the MHC at *CD74*.
- **Supplemental Fig. S8.** CIITA binding outside of the MHC at *B2M*.
- **Supplemental Fig. S9.** Comparison of CIITA BIs identified by ChIP-seq with ChIP-promoter array.
- **Supplemental Fig. S10.** Allelic differences in binding affinity and reporter gene expression involving rs11074938 investigated using the Jurkat cell line.
- **Supplemental Fig. S11.** Gating strategy for HLA class II surface expression on B cells.
- **Supplemental Fig. S12.** Illustration of the definition of a BI.

**Supplemental Table S1**

**Worksheet S1.1 sampleInfo**: Filenames and corresponding description for ChIP-seq datasets.

**Worksheet S1.2 mappingStat**: Statistics pertaining to mapping of ChIP-seq datasets.

**Worksheet S1.3 Correlation**: Pairwise-comparisons of binding affinities across ChIP-seq samples (DiffBind analysis).

**Worksheet S1.4 DHS**: Proportion of CIITA BIs that are within a transcriptionally accessible region (DHS)

**Worksheet S1.5 Known_CIITA_targets**: List of genes previously reported as CIITA targets.

**Worksheet S1.6 Gene_sets_CIITA_BIs**: Genes within 10kb 5' or 3' of a CIITA BI. Includes lists of genes where association found with CIITA BI in any one of B cells, naïve monocytes of IFN treated monocytes; in B cells; in naïve monocytes; and in interferon-treated monocytes. For analysis across cell types or for each cell type, genes associated with all CIITA BI are listed; or genes associated with CIITA BI overlapping RFX5 BI (CE-mark); genes associated with CIITA BI not overlapping RFX5; genes associated with any CIITA BI in the MHC; genes associated with any CIITA BI located outside the MHC; and genes associated with any CIITA BI located outside the MHC which are not previously described targets of CIITA (Worksheet S1.5). These different categories were used in Ingenuity Pathway Analysis (IPA) for which the results are given in worksheets S1.9-20.

**Worksheet S1.7 BindingIntervals_BI**: Detailed annotation of BIs and coincidence with datasets from ENCODE (includes DHS as transcriptionally accessible regions) and gene expression for B cells, naïve and treated monocytes.

**Worksheet S1.8 Notes**: notes to worksheet S1.7

**Worksheet S1.9 CIITA_pathways**: Pathways identified using IPA for genes associated with CIITA BIs in one or more of B cell, naïve monocyte or IFN treated monocytes.

**Worksheet S1.10 Bcell_pathways**: Pathways identified using IPA for genes associated with CIITA BIs in B cells.

**Worksheet S1.11 Mono_pathways**: Pathways identified using IPA for genes associated with CIITA BIs in naïve monocytes.

**Worksheet S1.12 MonoIFN_pathways**: Pathways identified using IPA for genes associated with CIITA BIs in IFN treated monocytes.

**Worksheet S1.13 CIITA_dis_funct**: Diseases and functions identified using IPA for genes associated with CIITA BIs in one or more of B cell, naïve monocyte or IFN treated monocytes.

**Worksheet S1.14 Bcell_dis_funct**: Diseases and functions identified using IPA for genes associated with CIITA BIs in B cells.

**Worksheet S1.15 Mono_dis_funct**: Diseases and functions identified using IPA for genes associated with CIITA BIs in naïve monocytes.

**Worksheet S1.16 MonoIFN_ dis_funct**: Diseases and functions identified using IPA for genes associated with CIITA BIs in IFN treated monocytes.

**Worksheet S1.17 CIITA_upstream_reg**: Upstream regulators identified using IPA for genes associated with CIITA BIs in one or more of B cell, naïve monocyte or IFN treated monocytes.

**Worksheet S1.18 Bcell_upstream_reg**: Upstream regulators identified using IPA for genes associated with CIITA BIs in B cells.

**Worksheet S1.19 Mono_upstream_reg**: Upstream regulators identified using IPA for genes associated with CIITA BIs in naïve monocytes.

**Worksheet S1.20 MonoIFN_upstream_reg**: Upstream regulators identified using IPA for genes associated with CIITA BIs in IFN treated monocytes.

**Worksheet S1.21 DAVID**: Biological themes from analysis using DAVID with enrichment of genes associated with a CIITA BI.

**Worksheet S1.22 Motif_enric_CIITA_nonRFX5:** Motif enrichment analysis of peak summits in CIITA BIs not overlapping RFX5 BI.

**Supplemental Methods**

**Primers used for determining enrichment after ChIP:**

| | |
|---|---|
| DMA_Fw | 5’- CCCATACCTTCTTGCCACAC -3’ |
| DMA_Rv | 5’- GACAAGGGCACCTATTGGAA -3’ |
| | |
| NFKBIL1_Fw | 5’- ACCTGTGTTGGGAAAAGAGC -3’ |
| NFKBIL1_Rv | 5’- TATCGTAGGATGGGGCAAGT -3’ |
| | |
| CD74_Fw | 5’- GTATTTCCAGCCTTTGTAGCTTTCAC -3’ |
| CD74_Rv | 5-‘ TGGAGAGGAATCTGATTCGTCC -3’ |

**Library preparation and sequencing for ChIP-Seq**

DNA was quantified using the High Sensitivity Qubit system (Life Tech) and the fragmentation profile was assessed using a DNA HS 2200 TapeStation chip (Agilent). ChIP libraries were prepared using the NEBNextTM DNA Sample Prep MasterMix Set 1, according to manufacturer's specifications, but with the following amendments. End repair was carried out using 5 ul of NEBNEXT End Repair Reaction Buffer and 0.5 ul of NEBNext End Repair Enzyme Mix. A-tail was carried out using 5 ul of NEBNEXT A-tail Reaction Buffer and 0.3 ul of NEBNext Klenow Fragment (3´→5´ exo-) Enzyme Mix. Ligation was carried out using 10 ul of NEBNEXT Quick Ligation Reaction Buffer and 0.5 ul of NEBNext Quick T4DNA Ligase Enzyme Mix. The NEBNext adapters were diluted 100-fold and the amount added was adjusted if less than 5 ng of ChIP material was available. No size selection was carried out. PCR was done using the following reaction mix with 18 cycles of PCR: DNA (36 µl), 5 X Phusion buffer (10 µl), Custom PE primer 1 (1 µl), Custom PE primer 2 (1 µl), dNTP mix (1.5µl), Phusion polymerase (0.5 µl). The clean-up after amplification was done at 1:0.85 (Ampure beads:DNA). The concentration of each library was determined by real-time PCR using Agilent qPCR Library Quantification Kit and a MX3005P instrument (Agilent). Sequencing was performed on an Illumina HiSeq2000 using 51-bp paired end reads.

**Differential binding across the various ChIP-seq datasets**

Pairwise-comparisons and clustering of datasets were performed using the DiffBind package. Read counts (obtained from BAM files of each ChIP-seq dataset) across a consensus set of peaks for the different datasets were derived using a trimmed mean of M-values (TMM). Read counts are representative of binding affinity for a given peak. Following normalisation of binding affinities datasets samples were re-clustered using default parameters.

**Primer and probe design for ddPCR**

| | |
|---|---|
| CD74_Tq_Fw | CTTTGGTGAAGCTGCCTTTT |
| CD74_Tq_Rv | CAGAAACAAGTGATGAGGGC |
| CD74_Tq_FAM | AGGCAGGTAGAAAGGCCAGCACGA |
| | |
| BCSL2_Tq_Fw | GCCACTCCCATTCCAAGAT |
| BCSL2_Tq_Rv | CAGCCATTTTGAGACCAGAA |
| BCSL2_Tq_FAM | CCCAGGCAACCGGCGTTCTCAATA |
| | |
| LYZ_Tq_Fw | CCTGTTTTCCACACAACTGA |
| LYZ_Tq_Rv | CTGCTAGGTCAGAGTGCTAG |
| LYZ_Tq_FAM | CCCAAACCACAAGGGGAAGAAGGAAGT |
| | |
| STAT1_Tq_Fw | AGACTCTGCGCAGGAAAG |
| STAT1_Tq_Rv | GAACAGCCGCGTCTAATTG |
| STAT1_Tq_FAM | ACTACCCGGCAGGAGAAAAGGCAG |
| | |
| B2M_Tq_Fw | AGTCTAGTGCATGCCTTCTT |
| B2M_Tq_Rv | ACCGTCACCTGTCTCCAA |
| B2M_Tq_FAM | TCTAACCTGGCACTGCGTCGCT |
| | |
| Rab4A2_Tq_Fw | GGGGTGAAATGTGTTGAATCT |
| Rab4A2_Tq_Rv | TTTGAAAAGGCAGCAGAGTT |
| Rab4A2_Tq_FAM | TCCCCATGTTGAAGCAGAGGCCA |
| | |
| Rab4A_Tq_Fw | GAGGATGGAGCCAAACTGAC |
| Rab4A_Tq_Rv | CTTGGCTAAGCTCCCAAGTG |
| Rab4A_Tq_YakimaYellow | ACTGGCATTTTCCACACAGTCCAGGT |

### Cell culture

Jurkat T cells were grown in RPMI 1640 (Sigma-Aldrich, Dorset UK) Supplemented with 2mM Glutamine (Sigma), 100U/ml penicillin (Sigma), 0.1mg/ml streptomycin (Sigma) and 10% FCS (Sigma) at 37°C in 5% $CO_2$ and harvested in mid log phase.

### Transient transfection and reporter gene analysis

DNA fragments corresponding to the putative regulatory intronic sequence spanning both alleles of rs11074938 in the *CIITA* gene were synthesized by PCR amplification of genomic DNA from two individuals (chr16: 11006438-11006614). PCR primer design introduced restriction sites to clone into *NotI/SacI* sites upstream of the luciferase gene in the -165KIP2 construct [1] derived from pGL3 Basic #E1751 (Promega, Madison, WI) generating pGL3-CIITA-rs11074938A and pGL3-CIITA-rs11074938G:

Fwd 5' -GCGGCCGCCTGATCACTGCTCCCCTGT-3',

Rev 5'- GAGCTCGAACTAGGGGCTTTTTCAGC-3'.

All constructs were verified by sequencing. $2x10^6$ Jurkat cells were transiently transfected using Lipofectamine LTX and PLUS reagent (Invitrogen) according to manufacturer's instructions. Cells were harvested after 24 hours and luciferase assays performed following the manufacturer's protocol. Four biological replicate transfections were performed. Firefly luciferase constructs were co-transfected with pRL-TK (Promega) to allow normalisation of transfection efficiencies. Two independent endotoxin-free preparations of all constructs were analysed in transfection experiments.

### References

1. Dauphinot L, De Oliveira C, Melot T, Sevenet N, Thomas V, Weissman BE, Delattre O: **Analysis of the expression of cell cycle regulators in Ewing cell lines: EWS-FLI-1 modulates p57KIP2and c-Myc expression.** *Oncogene* 2001, **20:**3258-3265.

A

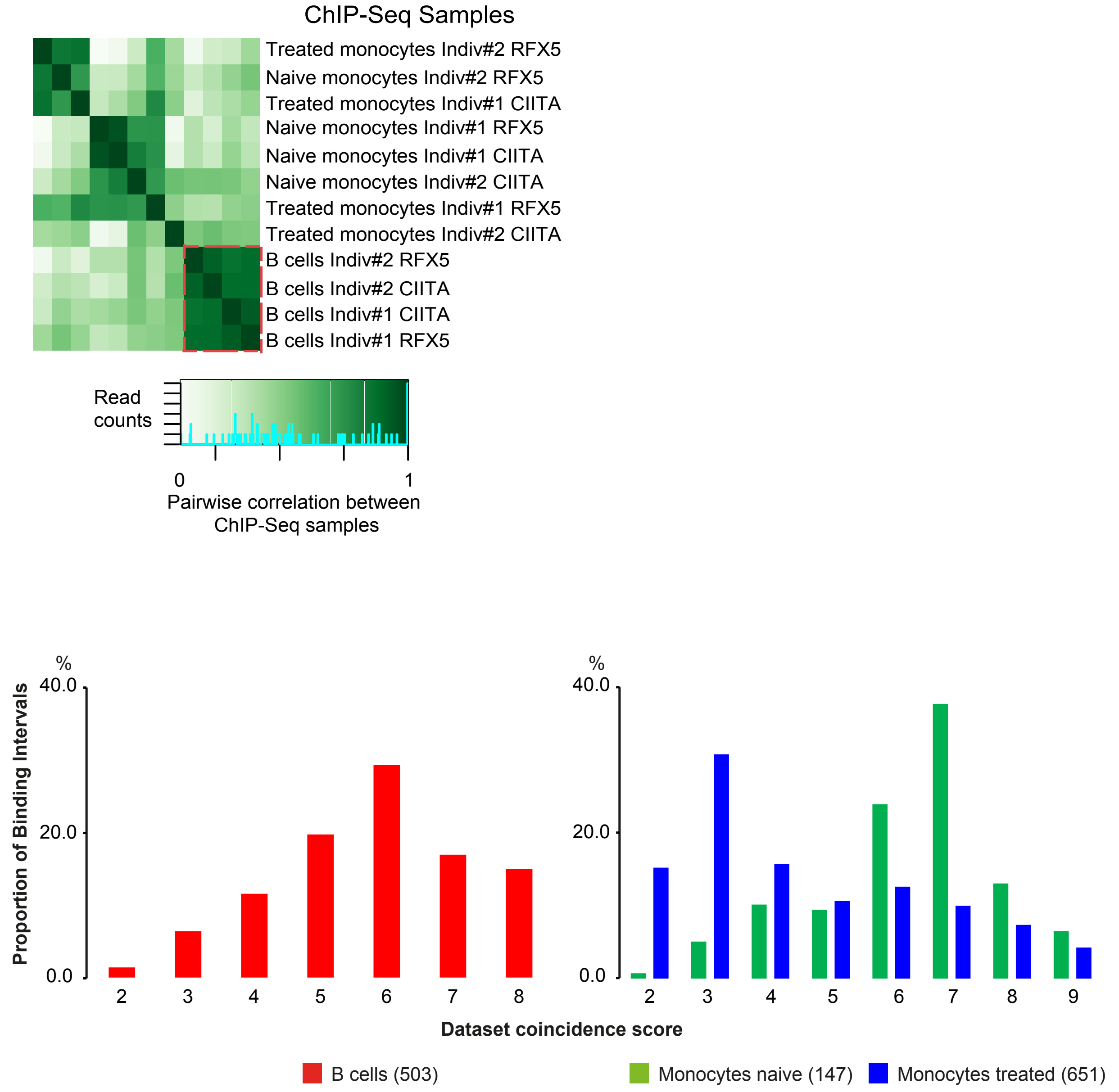


**Supplemental Figure S1. CIITA and RFX5 binding affinities and coincidence with components of CIITA enhanceosome.** (A) CIITA and RFX5 binding affinities form distinct signatures for B cells and monocytes. Clustering of pairwise correlations between individual ChIP-seq samples partitioned B cells and monocytes into two distinct groupings. Correlation coefficients (Spearman) between individual samples within each cell type grouping are 0.9 for B cells and 0.3 - 0.9 for monocytes. Pairwise comparisons between the binding affinities of B cell, naive and treated monocyte groupings were also performed. Correlation coefficients (Spearman) are for between: naive and treated monocytes 0.76; B cells and naive monocytes 0.48; B cells and treated monocytes 0.32. (B) CIITA and RFX5 bind in genomic locations that are accessible to other transcription factors including constituents of the CIITA Enhanceosome. Data Coincidence Scores (DCS) indicate number of datasets including data from the ENCODE consortium that coincide with CIITA and RFX5 Binding Intervals (BIs) in both B cells and monocytes. In treated monocytes, CIITA binding showed a relatively lower extent of coincidence. Data from the ENCODE project used for these comparisons had been generated with cells in a naïve state, accounting for the lower concordance between this and our data from treated monocytes. Datasets used in coincidence analysis for B cells: CIITA and RFX5 (our data from primary B cells); CREB1, NF-Y and RFX5 in GM12878 LCL (ENCODE); DNase I HS-sites (DHS) in primary B cells (ENCODE). For monocytes: CIITA and RFX5 (our data from naive and treated monocytes); ATF1, CREB1, NF-Y and RFX5 in K562 (ENCODE); DHS in primary monocytes (ENCODE). Number of BIs in each dataset shown within parentheses.

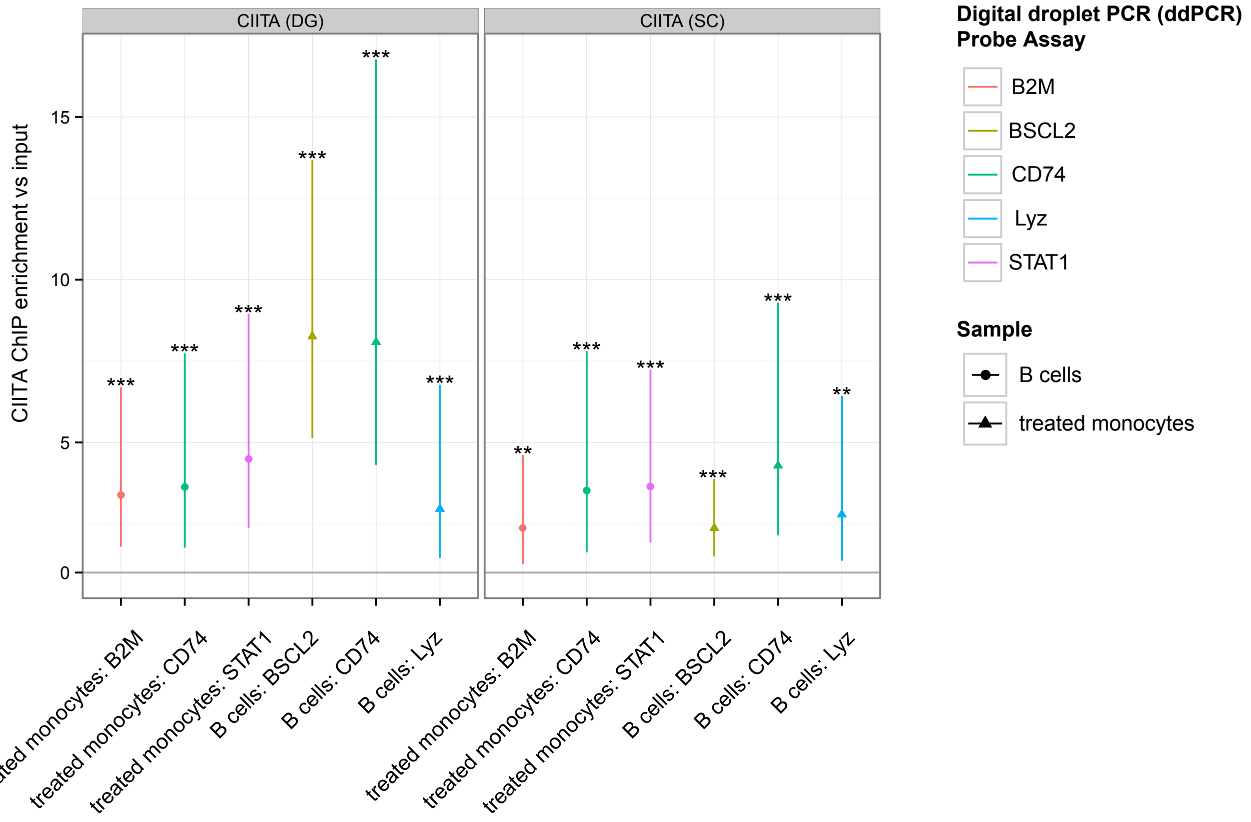


**Supplemental Figure S2. Validation of CIITA ChIP.** Enrichment of DNA from CIITA ChIP compared to that of corresponding input as determined using ddPCR for chromatin prepared from monocytes and B cells isolated from an independent leukocyte cone (third individual donor). Antibodies CIITA (DG) and CIITA (SC) were obtained from Diagenode and SantaCruz respectively. Probe assays were designed spanning CIITA BI identified by ChIP-seq in the region of the following genes: *B2M* (CIITA BI in treated monocytes); *STAT1* (CIITA BI in treated monocytes); *BSCL2* (CIITA BI in B cells); *LYZ* (CIITA BI in B cells); and *CD74* (CIITA BI in B cells and treated monocytes). Rescaled enrichment estimates are shown with 95% confidence intervals. Significance of enrichment based on Poisson test are shown *** P <0.001, ** P <0.01.

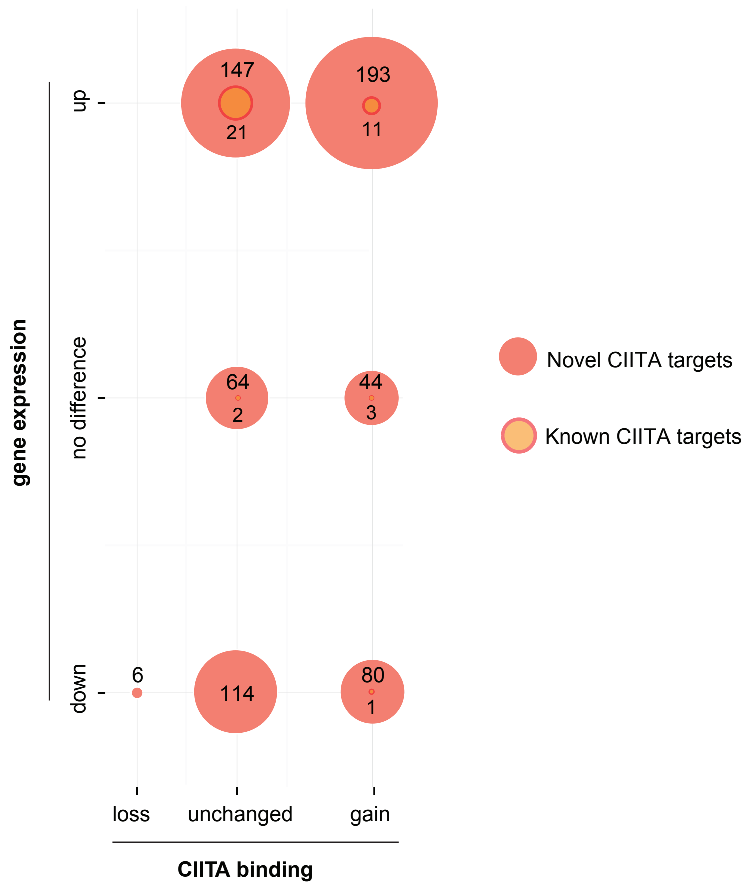


**Supplemental Fig. S3. CIITA binding and gene expression in monocytes.** Concordance of differential gene expression between IFNγ treated and naive monocytes (up, down or no difference) and corresponding change, if any in CIITA occupancy. Numbers correspond to novel and known targets of CIITA respectively. Differentially expressed monocyte genes defined using limma model fitting with adjusted P<0.01 [16].

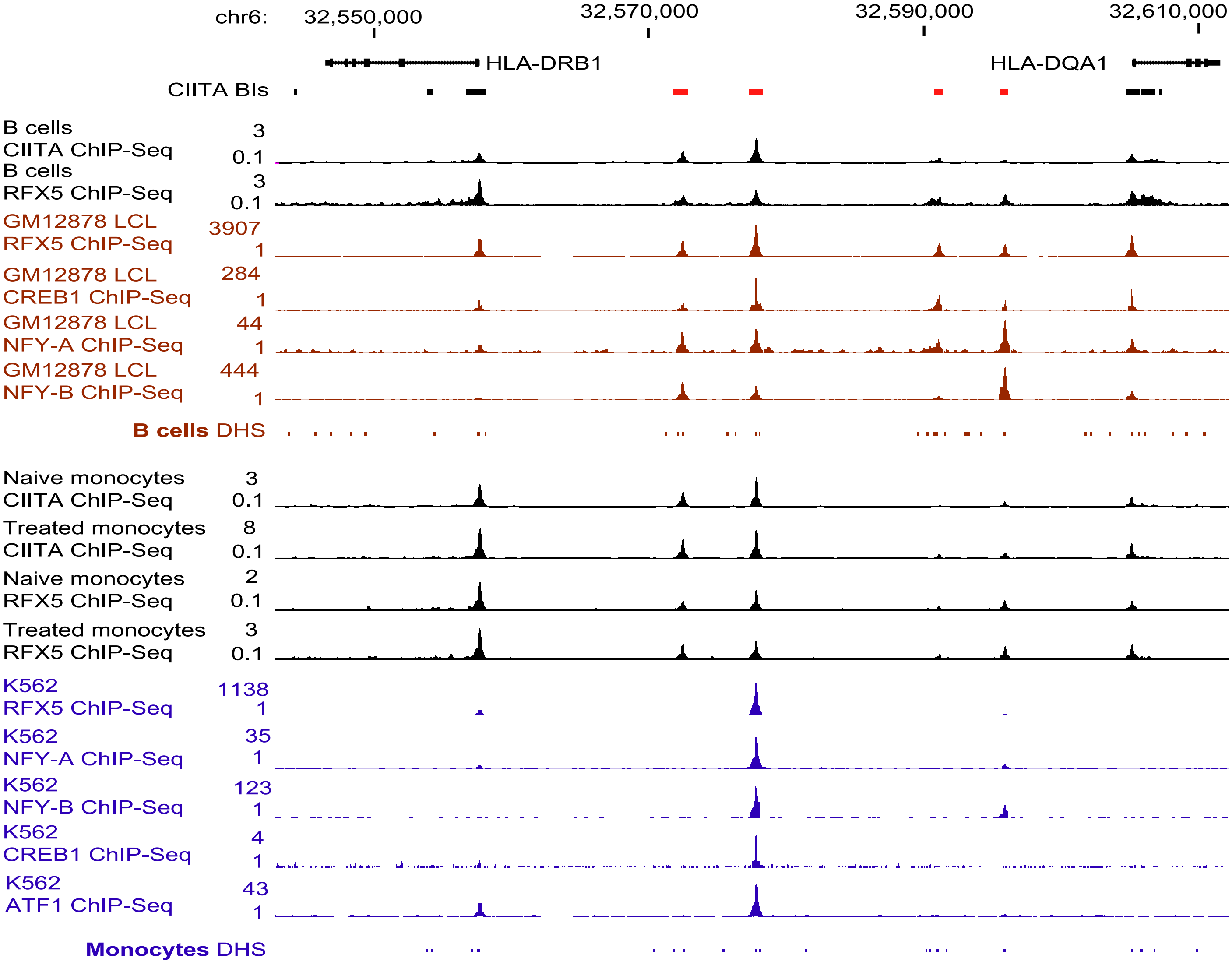


**Supplemental Figure S4. Intergenic CIITA binding in the interval between *HLA-DRB1* and *HLA-DQA1* within the MHC Class II region.** ChIP-seq data shown for CIITA and RFX5 in relation to ENCODE data for GM12878 and K562 cells. Four distal CIITA BIs in the interval between *HLA-DRB1* and *HLA-DQA1* highlighted (in red).

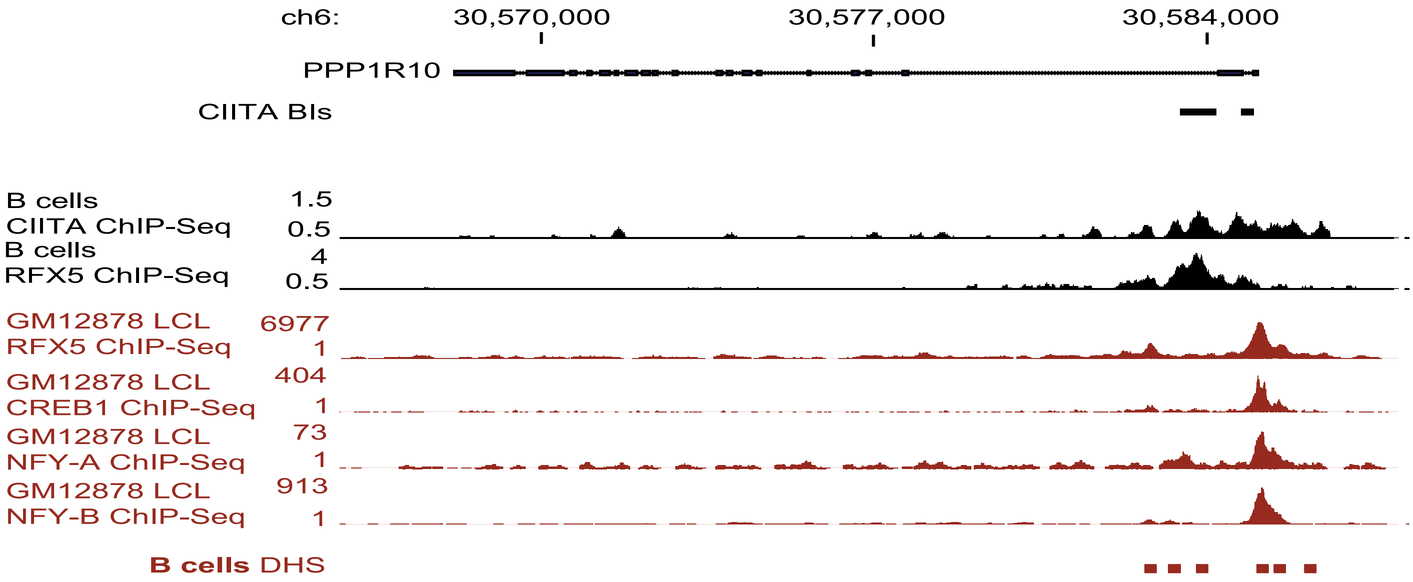


**Supplemental Figure S5. CIITA binding within the MHC Class I region at *PPP1R10*.** ChIP-seq data shown for CIITA and RFX5 in B cells together with ENCODE data for GM12878. *PPP1R10* encodes the protein phosphatase I regulator PNUTS.

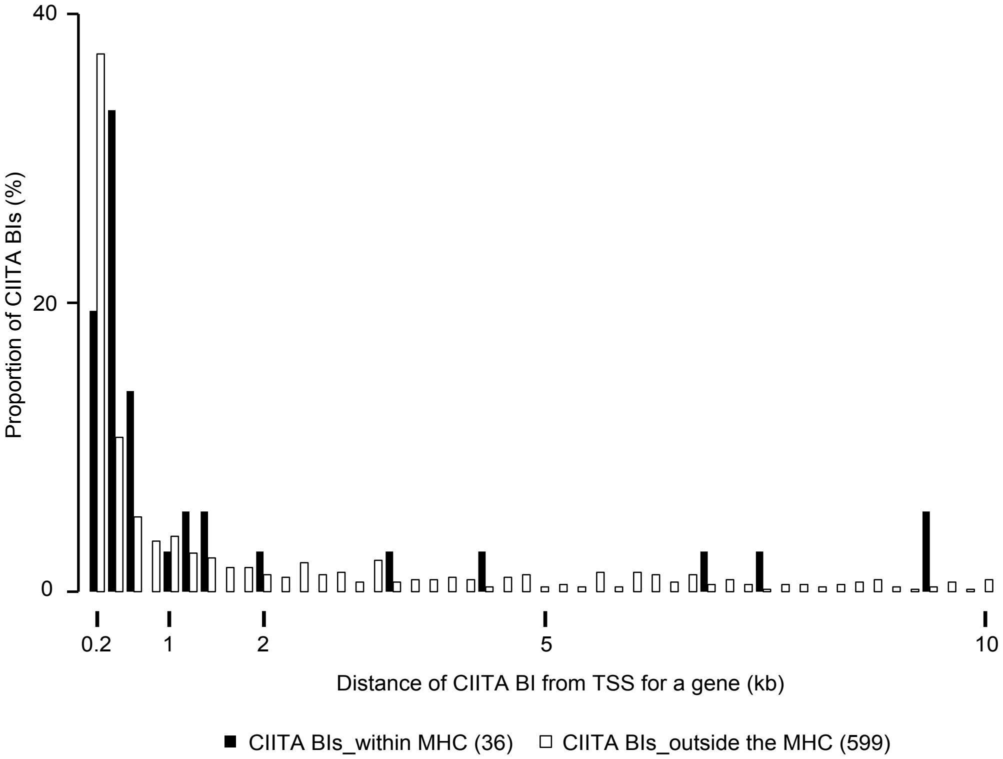


**Supplemental Figure S6. Distribution of CIITA BIs localised within and outside of the MHC region in relation to relative distance from the TSS.** Number of CIITA BIs within and outside the MHC shown in parentheses.

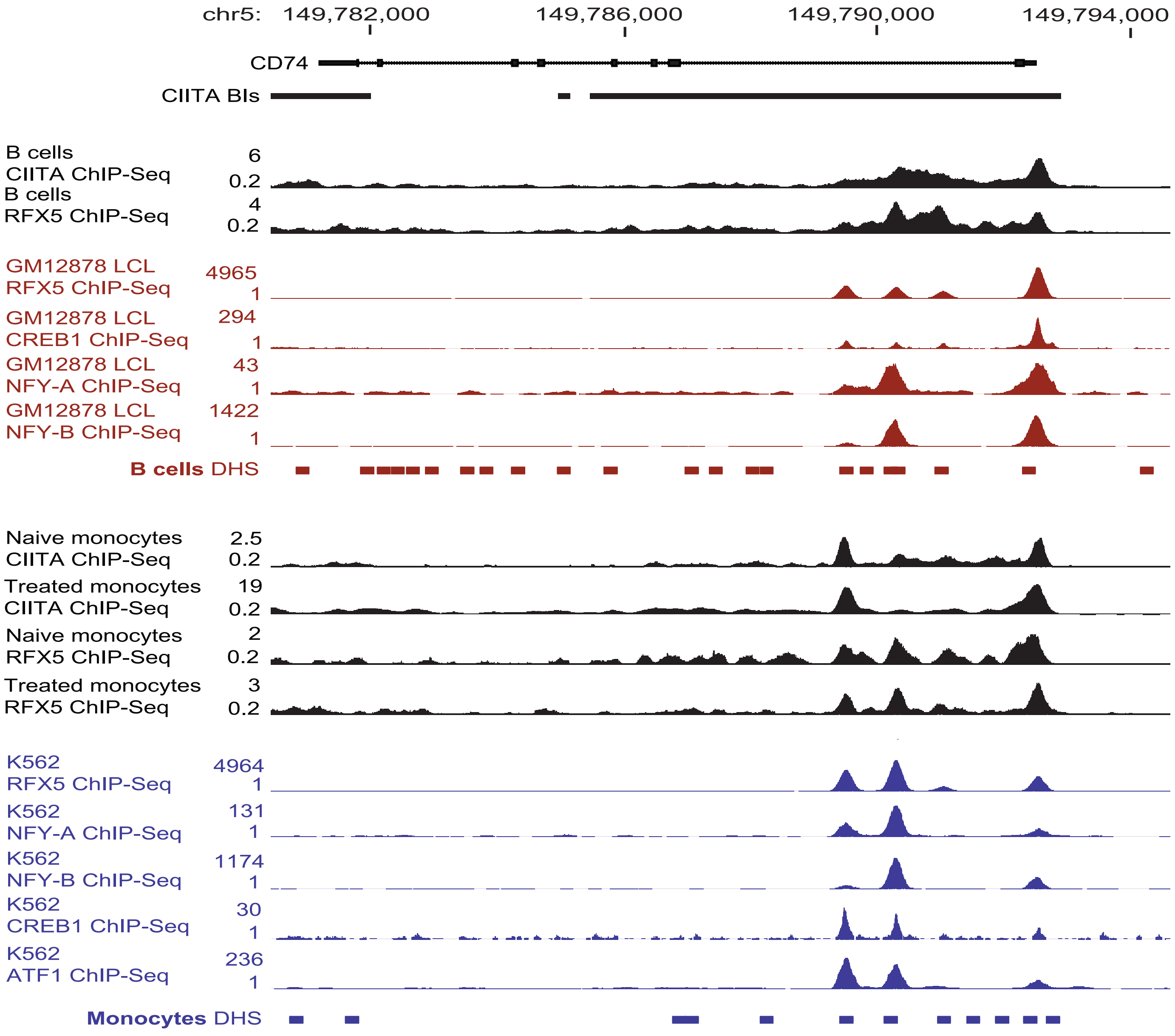


**Supplemental Figure S7. CIITA binding outside of the MHC at *CD74*.** ChIP-seq data shown for CIITA and RFX5 in relation to ENCODE data for GM12878 and K562 cells.

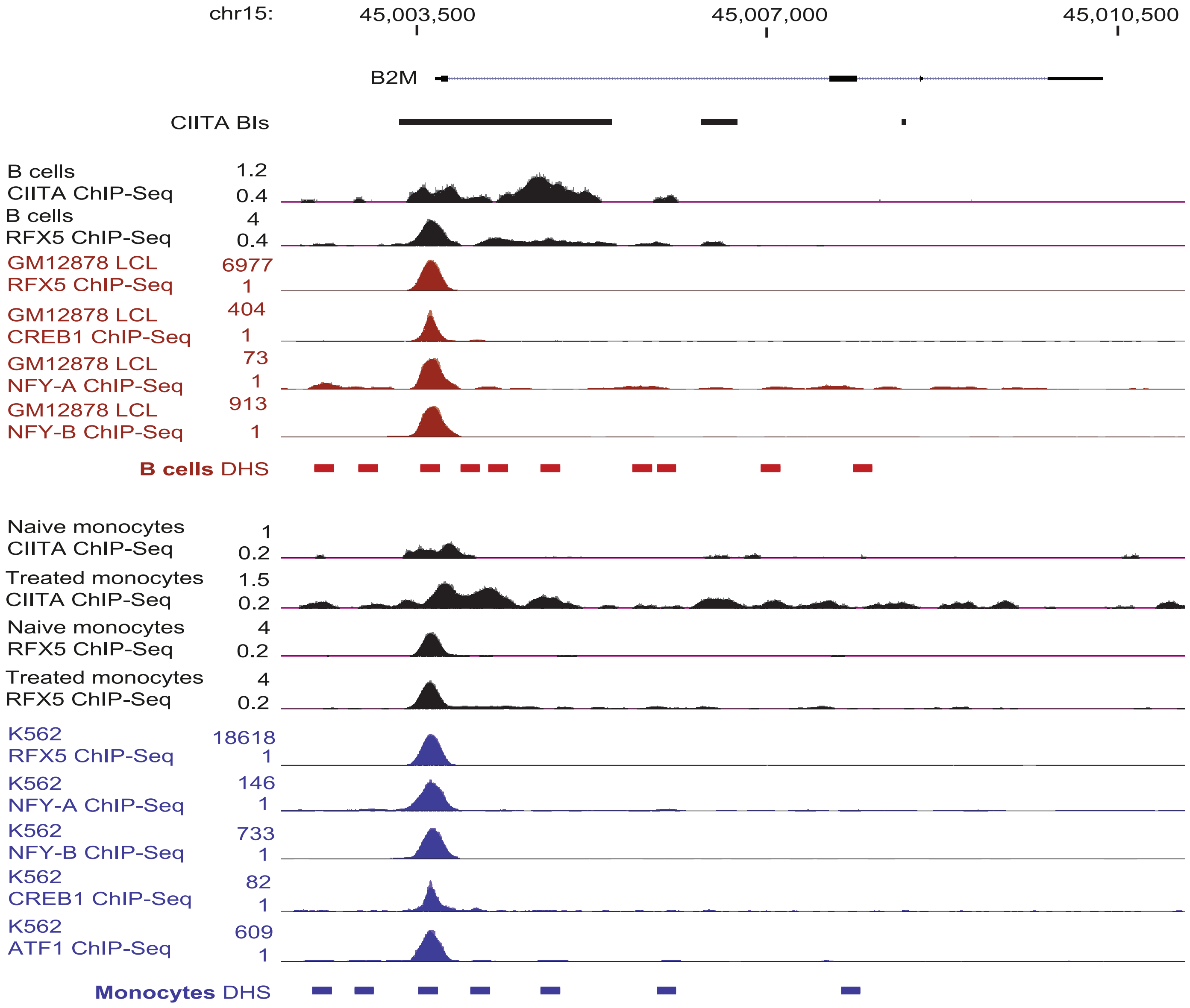


**Supplemental Figure S8. CIITA binding outside of the MHC at *B2M*.** ChIP-seq data shown for CIITA and RFX5 in relation to ENCODE data for GM12878 and K562 cells.

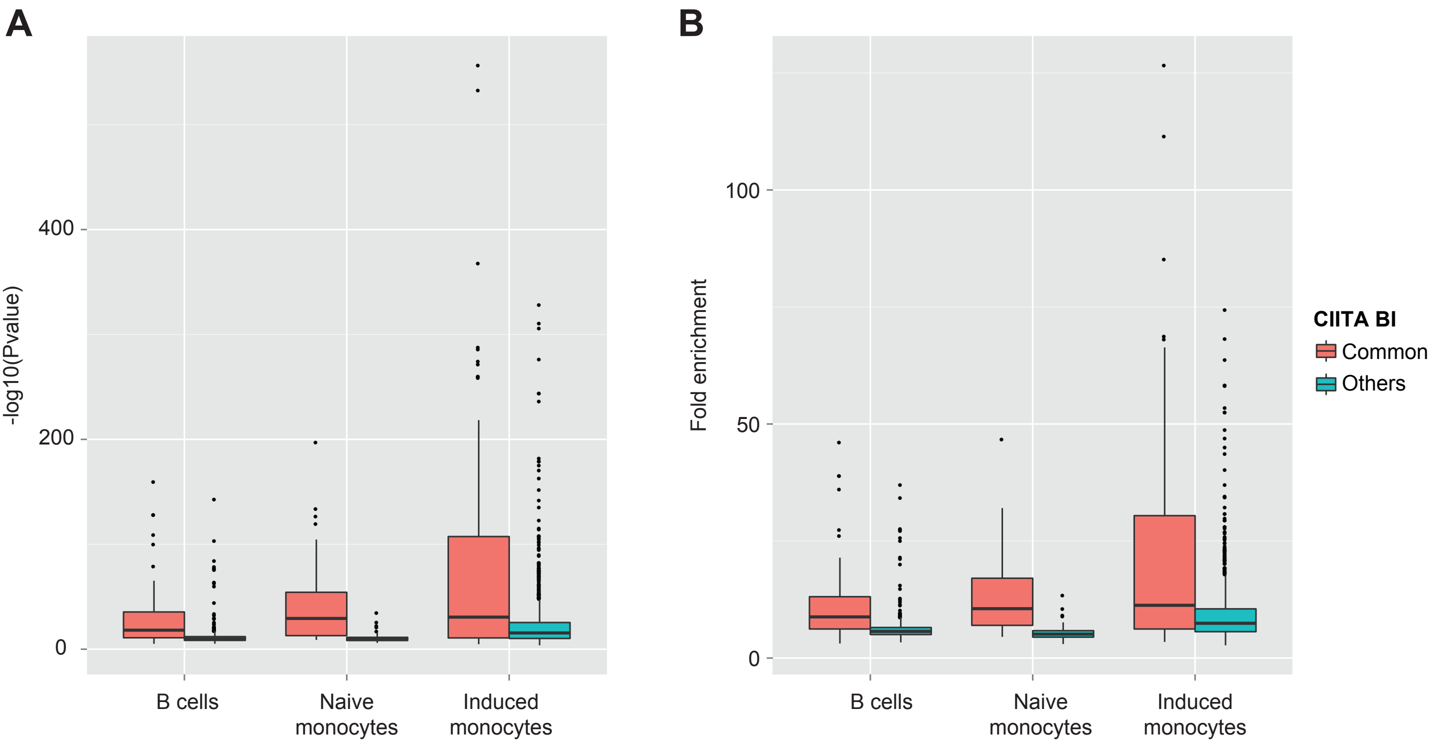


**Supplemental Fig. S9. Comparison of CIITA BIs identified by ChIP-seq with ChIP-promoter array.** (A) -log10(pvalue) and (B) Fold enrichment compared to corresponding input experiments. Data generated by MAC2 peak-calling software and presented as box plots for CIITA BIs (-4kb to 1kb relative to TSS) identified by both methods (denoted as 'Common') and those not identified by the promoter array ('Others'). Common CIITA BI comprise 19 genes previously reported as confirmed CIITA targets by ChIP-promoter array [8] (*HLA-DMA*, *HLA-DMB*, *HLA-DOA*, *HLA-DOB*, *HLA-DPA1*, *HLA-DPB1*, *HLA-DQA1*, *HLA-DQB1*, *HLA-DRA*, *HLA-DRB1*, *HLA-DRB5*, *CD74*, *PSMD3*, *MYBPC2*, *RAB4B*, *RFX5*, *TPP1*, *TRIM26* and *ZNF672*) for which we find CIITA BIs -4kb to 1kb relative to the TSS (criteria used by authors). Median values for fold enrichment in B cells (Common 8.51, Others 5.63), naive monocytes (Common 13, Others 5.11), treated monocytes (Common 27.55, Others 7.3); -log10(pvalue) in B cells (Common 18.41, Others 9.83), naive monocytes (Common 29.29, Others 9.49), treated monocytes (Common 30.56, Others 15.48).

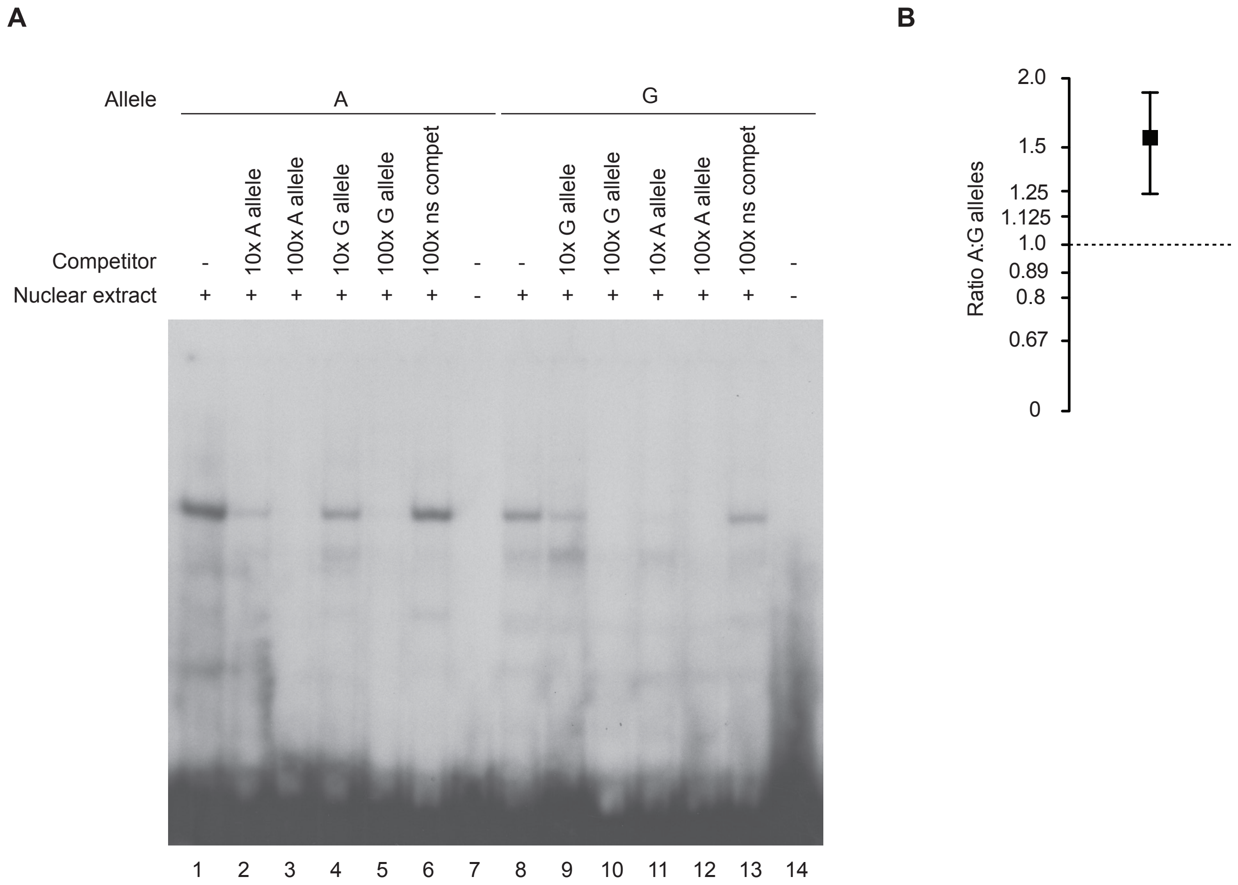


**Supplemental Fig. S10. Allelic differences in binding affinity and reporter gene expression involving rs11074938 investigated using the Jurkat cell line**. (A) EMSA using crude nuclear extracts prepared from Jurkat cells incubated with radiolabelled probes corresponding to the two different alleles for rs11074938 are shown, either for the A allele (lanes 1-7) or G allele (lanes 8-14). To investigate specificity, molar excess of unlabelled probe corresponding to the A allele (lanes 2,3,11,12), G allele (lanes 4,5,9,10) or a non-specific competitor (lanes 6 & 13) was also included in binding reactions as shown. (B) Allele-specific differences in reporter gene expression for rs11074938. Reporter gene constructs containing the intronic sequence for CIITA spanning rs11074938 (177 bp fragment chr16: 11006438-11006614) cloned into the -165KIP2 reporter construct and transiently transfected into Jurkat cells showed higher expression associated with the rs11074938 A allele, with a mean ratio between the A and G alleles of 1.56 (95% confidence intervals 1.24-1.89; paired t test P= 0.022). Mean and 95% confidence intervals shown (n=8).

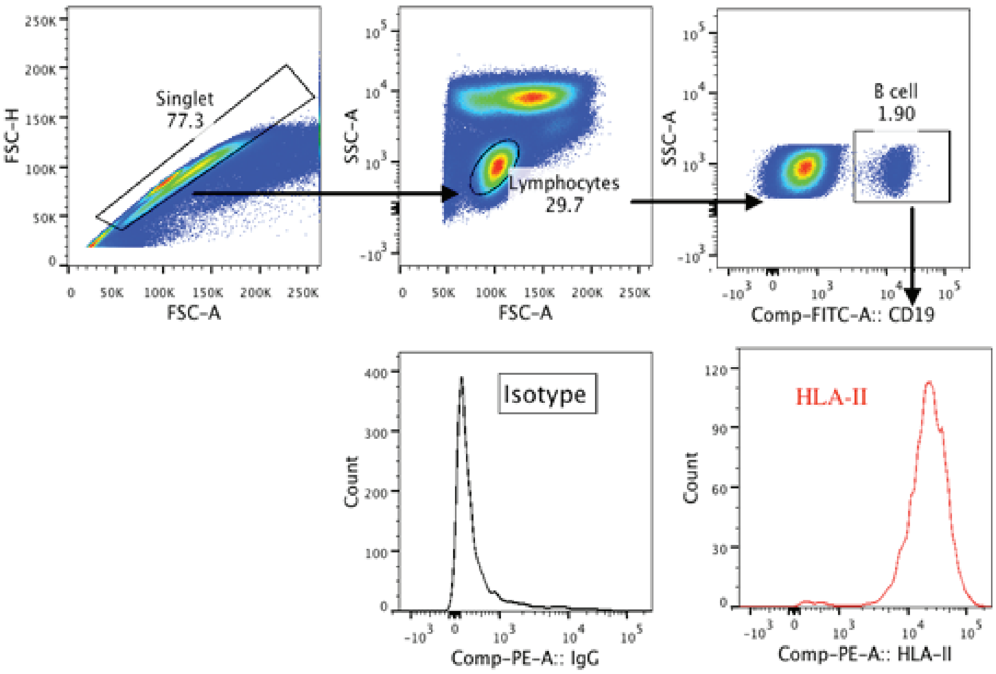


**Supplemental Figure S11. Gating strategy for HLA class II surface expression on B cells.** Lysed whole blood was stained as described and data acquired on a BD FACSCanto. Singlets and lymphocytes were gated on light scatter properties as shown and CD19+ lymphocytes were assessed for their HLA-II signal. Data from a single individual ("HLA-II") and for comparison, the staining from an isotype control ("isotype") is shown.

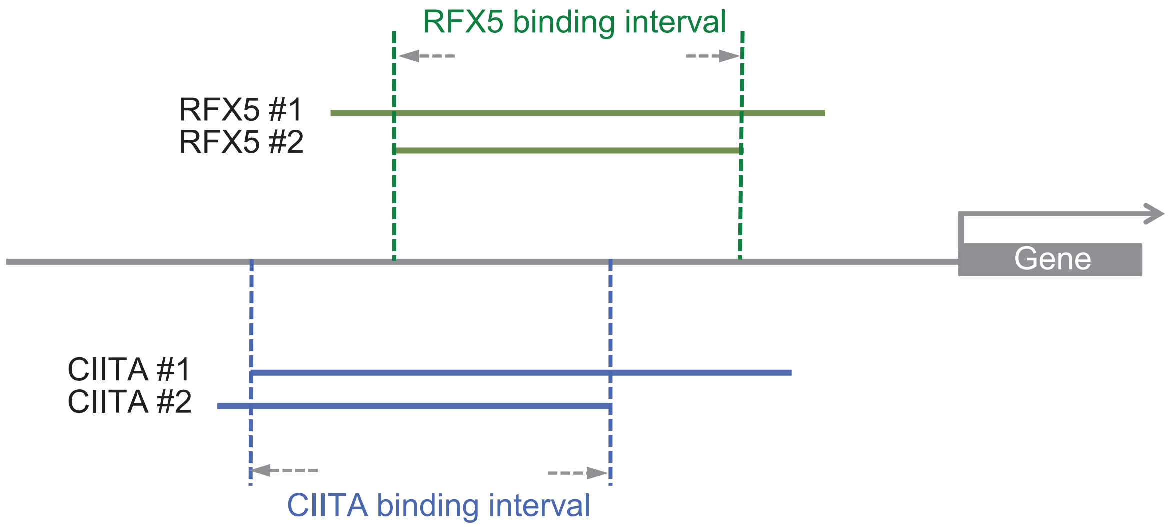


**Supplemental Fig. S12. Illustration of the definition of a BI.** A CIITA or RFX5 BI was identified if there were corresponding peaks that intersected across both individuals. A CIITA BI was further identified to be a CE-mark if there was overlap with at least one RFX5 peak from either individual. All CE-marks are also CIITA BIs but not all E-marks are necessarily RFX5 BIs.
